# Curcumin Protects Mouse Spermatogonia from Triptolide-Induced Injury Through Modulation of Ferroptosis-Related Pathways

**DOI:** 10.3390/biology15131019

**Published:** 2026-06-26

**Authors:** Chenyang Wang, Pengfei Zhang, Xuyang Liu, Mingxing Li, Long Chen, Qianqian Yang, Yulin Huang

**Affiliations:** 1 School of Basic Medicine, Guangxi University of Chinese Medicine, Nanning 530200, China; wangcy920@163.com (C.W.); liuxuyang0301@163.com (X.L.); lmx4986@163.com (M.L.); chenl2015@gxtcmu.edu.cn (L.C.); 18820301970@139.com (Q.Y.); 2 Guangxi Key Laboratory of Integrated Traditional Chinese and Western Medicine Translational Medicine for High-Incidence Infectious Diseases, Nanning 530200, China; 3 College of Animal Science and Technology, Guangxi University, Nanning 530004, China; pengfeizhang2016@163.com; 4 Key Laboratory of Characteristic Experimental Animal Model of Guangxi, Nanning 530200, China

**Keywords:** curcumin, triptolide, spermatogonia, proteomics, ferroptosis, male reproductive toxicity, testicular protection

## Abstract

Triptolide (TP) is an effective anti-inflammatory and immunosuppressive agent, yet its clinical application is constrained by significant male reproductive toxicity. Curcumin, a natural antioxidant, exhibits protective effects; however, whether it protects against TP-induced damage during mouse spermatogenesis and the underlying mechanisms remain incompletely understood. Methods: Proteomic analysis was performed to investigate the protective mechanism of curcumin in mouse GC-1 cells, followed by multiple validation assays including CCK-8 assay, apoptosis detection, measurement of reactive oxygen species (ROS), glutathione (GSH), malondialdehyde (MDA), and Fe^2+^ levels, quantitative polymerase chain reaction (qPCR), Western blotting (WB), hematoxylin-eosin (HE) staining, and immunofluorescence. Results: Proteomic analysis revealed that curcumin primarily ameliorated TP-induced damage in mouse spermatogonia by modulating ferroptosis-related pathways. Curcumin elevated GSH levels; reduced MDA, ROS, and Fe^2+^ levels; alleviated lipid peroxidation; and regulated ferroptosis-related pathways in both TP-induced GC-1 cells and testicular tissue. These effects were associated with upregulation of the mRNA and protein expression of *Nrf2*, *Gclc*, and *Map1lc3a* and downregulation of *Tfrc* and *Dmt1*. Collectively, these findings demonstrate the protective effect of curcumin against TP-induced spermatogonial damage. Conclusions: Curcumin regulated ferroptosis-related pathways by modulating the expression of *Nrf2*, *Gclc*, *Map1lc3a*, *Tfrc*, and *Dmt1*, thereby significantly ameliorating TP-induced damage in mouse spermatogonia.

## Simple Summary

Triptolide, an effective anti-cancer and immunosuppressive drug, causes male reproductive toxicity. Curcumin, a natural antioxidant, was investigated for its protective effects against triptolide-induced spermatogonial injury using GC-1 cells and a mouse model. Curcumin significantly reduced cell damage and preserved testicular architecture. Mechanistically, curcumin may modulate ferroptosis—an iron-dependent, lipid peroxidation-driven cell death—by altering the expression of key genes including *Nrf2*, *Gclc*, *Map1lc3a*, *Tfrc*, and *Dmt1*. These findings suggest that curcumin co-administration may preserve male fertility during triptolide therapy, offering a step toward safer clinical use.

## 1. Introduction

Triptolide (TP), one of the primary active components of the traditional Chinese medicinal herb *Tripterygium wilfordii*, is also the main active ingredient of Tripterygium tablets and Tripterygium glycosides. TP exhibits a range of pharmacological activities, including anti-cancer, anti-inflammatory, and immunosuppressive effects, and is clinically used to treat systemic lupus erythematosus and rheumatoid arthritis [1,2,3]. However, the clinical application of TP is limited by its severe adverse effects, particularly its pronounced toxicity to the reproductive system. A previous study reported that when male SD rats receiving daily oral administration of triptolide were mated with untreated females, the mating success rate was significantly reduced, and the pregnancy rate of the female rats was markedly decreased. After drug withdrawal, the pregnancy rate gradually recovered over time but remained lower than that of the control group until week 14 [4]. Numerous studies have shown that TP can cause reproductive system damage through pathways involving oxidative stress, apoptosis, and DNA damage [5,6]. Specifically, TP inhibits spermatogenic cell proliferation by activating apoptotic pathways and increasing reactive oxygen species (ROS) levels, thereby inducing oxidative stress [7].

Ferroptosis is an oxidative, iron-dependent form of cell death distinct from apoptosis, autophagy, and other cell death pathways [8]. Substantial evidence indicates that TP can induce ferroptosis. For instance, TP triggers ferroptosis in hepatocytes, characterized by iron accumulation and lipid peroxidation. Enhancing the expression of nuclear factor erythroid 2-related factor 2 (Nrf2) significantly reduces TP-induced ferroptosis. During TP-induced liver injury in mice, TP may directly bind to Nrf2 and promote its degradation via the ubiquitin-proteasome pathway, thereby inhibiting Nrf2 expression. Furthermore, in hepatocytes, TP decreases ferritin heavy chain 1 (FTH1) levels and increases transferrin receptor 1 (TFRC) levels, thereby facilitating iron uptake [9]. Studies have shown that TP causes significant testicular structural damage and spermatogenic defects, promotes the generation of ROS and malondialdehyde (MDA), and reduces glutathione (GSH) levels. TP also triggers polyubiquitination of glutathione peroxidase 4 (GPX4), promoting its ubiquitin-mediated degradation, thereby stimulating ferroptosis in mouse spermatocytes (GC-2) and exacerbating DNA damage [10]. Therefore, TP may induce spermatogonial damage by promoting ferroptosis.

Curcumin, primarily extracted from the traditional Chinese medicinal herb turmeric (Curcuma longa), is the most bioactive low-molecular-weight phenolic compound in turmeric and is widely recognized as a potent antioxidant. It counteracts oxidative stress through multiple mechanisms, including scavenging free radicals, activating antioxidant enzymes, and inhibiting lipid peroxidation [11]. Several studies have demonstrated the protective effect of curcumin against oxidative damage in male mouse spermatogenic cells and testicular tissue. For example, Poojary et al. [12] reported that curcumin nanocrystals improved seminiferous tubule structure and sperm functional capacity while reducing testicular cell apoptosis in mice following cyclophosphamide induction. Similarly, Bayramova et al. [13] found that curcumin treatment ameliorated carbon tetrachloride-induced testicular morphological damage and testosterone synthesis impairment, decreased the expression of the apoptosis-related protein caspase-3, and protected against carbon tetrachloride-induced oxidative damage in testicular tissue. Concurrently, curcumin protects the heart against ischemia/reperfusion injury by regulating the Sirt1/AKT/FoxO3a signaling pathway to inhibit ferroptosis, thereby preserving cardiac function [14,15]. Moreover, curcumin inhibits ferroptosis and alleviates patulin (PAT)-induced acute kidney injury by activating the p62/Keap1/Nrf2 pathway and upregulating antioxidant stress factors [16]. Recent studies have demonstrated that curcumin exerts protection against cadmium-induced testicular ferroptosis [17] and attenuation of testicular ischemia–reperfusion injury via GPX4 upregulation [18]. These findings support the hypothesis that curcumin may similarly counteract TP-induced spermatogonial ferroptosis.

Proteomic methods provide a robust foundation for studying the spatiotemporal dynamics of proteins and gaining deeper insights into biological functional regulatory networks [19]. Moreover, the DIA mode eliminates the stochasticity of data-dependent acquisition (DDA) and, combined with precise four-dimensional coordinate alignment, renders quantitative results across large sample sets more reliable and stable, exhibiting higher scanning speed and detection sensitivity compared with classical proteomics [20]. The DIA proteomics approach enables effective identification of protein expression changes under different cellular states, allowing comprehensive and dynamic analysis of protein expression variations [21]. This approach is highly valuable for investigating the molecular mechanisms underlying the protective effect of curcumin against TP-induced spermatogonial damage.

The GC-1 spg cell line, whose morphology falls between that of type B spermatogonia and primary spermatocytes, is widely used as an in vitro model to investigate early spermatogenesis and the function of genes involved in this process [22]. GC-1 cells are a well-characterized, SV40-immortalized mouse germ cell line that retains key features of undifferentiated spermatogonia. While they do not fully recapitulate the complexity of primary spermatogonia or the testicular niche, they are widely used for studying spermatogonial responses to toxicants and potential protective agents, including triptolide and curcumin. To date, few studies have reported the protective role of curcumin in TP-induced spermatogonial damage. Therefore, the present study established a TP-induced damage model in GC-1 cells and employed DIA proteomics to explore the effects of TP on GC-1 cells and the protective mechanism of curcumin against TP-induced damage in mouse spermatogonia. The findings aim to provide a reference for reducing male reproductive injury and mitigating apoptosis during TP therapy, to offer a theoretical basis for the use of curcumin to alleviate ferroptosis-induced reproductive damage, and to suggest new directions for the protective application of curcumin in reproductive injury.

## 2. Materials and Methods

### 2.1. Cell Culture and Treatment

GC-1 cells were cultured in high-glucose DMEM medium (BDBIO, Hangzhou, China) supplemented with 10% fetal bovine serum (Procell, Wuhan, China) and 1% penicillin-streptomycin (BDBIO, Hangzhou, China), using culture dishes maintained in a humidified incubator at 37 °C with 5% CO_2_. The control group (Con) was treated with 0.1% DMSO solution; the triptolide group (TP) was treated with 200 nM triptolide (Sparkjade, Qinan, China); the low-dose curcumin group (Cur-L) was treated with 2 μM curcumin (MCE, Shanghai, China) plus 200 nM triptolide; the medium-dose curcumin group (Cur-M) was treated with 4 μM curcumin plus 200 nM triptolide; the high-dose curcumin group (Cur-H) was treated with 6 μM curcumin plus 200 nM triptolide. Among these, the Con, TP, and Cur-M groups were used for proteomic analysis, while a ferroptosis-inhibitor group (TP+Fer-1, treated with 200 nM triptolide plus 2.5 μM ferrostatin-1) was additionally included for subsequent validation of ferroptosis-related pathways. The basis for the selection of triptolide (TP) and curcumin doses is detailed in Supplementary File S1. All groups were subjected to drug treatment for 24 h.

### 2.2. Animal Grouping and Treatment

Fifty 6-week-old male Kunming mice (Hunan Silaike Jingda Laboratory Animal Co., Ltd., Changsha, China) were acclimated for 5 days. All mice were housed in an environmentally controlled facility equipped with a standardized lighting and ventilation system. Throughout the experiment, mice were housed under environmental conditions with a temperature of 23 ± 1 °C and a relative humidity of 60–70%. Following each drug administration, the animals were placed in an environment at 25 °C and observed for 30 min. The mean initial body weight of all animals was 34.19 ± 1.84 g (mean ± SD), with no significant differences among the groups (*p* > 0.05). The animals were randomly divided into the following groups: the negative control group (Con), receiving intragastric administration of normal saline once daily; the triptolide group (TP), receiving intragastric administration of 0.4 mg/kg TP once daily; the curcumin low-dose group (Cur-L), receiving intraperitoneal injection of 50 mg/kg CUR along with intragastric administration of 0.4 mg/kg TP once daily; the curcumin medium-dose group (Cur-M), receiving intraperitoneal injection of 100 mg/kg CUR along with intragastric administration of 0.4 mg/kg TP once daily; and the curcumin high-dose group (Cur-H), receiving intraperitoneal injection of 200 mg/kg CUR along with intragastric administration of 0.4 mg/kg TP once daily. Each group comprised 10 mice. All mice received the respective drug interventions for 21 days, and body weight was measured every 5 days. On the final day of the experiment, all mice were fasted for 12 h. After euthanasia, the testes and epididymides were removed and weighed. From each animal, one testis was fixed in 4% paraformaldehyde, and the contralateral testis was frozen at −80 °C for subsequent protein and RNA extraction.

### 2.3. CCK-8 Assay

GC-1 cells were seeded in 96-well plates at a density of 5 × 10^4^ cells/mL and cultured for 24 h, followed by drug treatment for another 24 h. Subsequently, 20 μL of CCK8 reagent (APExBIO, Shanghai, China) was added to each well, and the plates were incubated at 37 °C for 2 h. Absorbance was then measured at 450 nm using a microplate reader (Tecan, Shanghai, China).

### 2.4. Annexin V-FITC/PI Apoptosis Assay

GC-1 cells were seeded at a density of 5 × 10^5^ cells per well in 6-well plates, cultured for 24 h, and then subjected to drug treatment for an additional 24 h. After treatment, cells were collected, centrifuged at 1000× *g* for 5 min, and the supernatant was discarded. The cell pellet was washed once with PBS. Subsequently, an Annexin V-FITC apoptosis detection kit (Beyotime, Shanghai, China) was used. Briefly, 195 μL of Annexin V-FITC binding buffer was added to gently resuspend the cells, followed by the addition of 5 μL Annexin V-FITC and gentle mixing. Then, 10 μL of propidium iodide staining solution was added, and the mixture was gently mixed. The samples were incubated at room temperature (20–25 °C) in the dark for 15 min, and then analyzed by flow cytometry.

### 2.5. Protein Extraction, Reduction/Alkylation, and Enzymatic Digestion

The samples were processed using a protein preparation kit (Omicsolution, Shanghai, China). To each GC-1 cell sample, 300 μL of lysis buffer (P0013B, Beyotime, Shanghai, China) was added, followed by sonication in an ice-water bath for 20 min. The lysate was then centrifuged at 12,000 rpm for 10 min at 4 °C, and the supernatant was transferred to a fresh microcentrifuge tube. The extracted protein was quantified using a BCA assay. Subsequently, 30 μg of protein was mixed with 20 μL of reagent A and incubated at 95 °C for 5 min with agitation at 1000 rpm. After cooling to room temperature, 15 μL of trypsin (reagent B) was added, and the mixture was digested at 37 °C for 2 h with shaking at 1000 rpm. The enzymatic reaction was terminated by adding 55 μL of reagent C and mixing thoroughly. The entire sample was then loaded onto a desalting column for desalting. After desalting, the sample was concentrated using a vacuum centrifugal concentrator under freezing conditions, reconstituted in an appropriate solvent, and prepared for subsequent instrumental analysis.

### 2.6. NanoLC-MS/MS Detection

After reduction (alkylation) and proteolytic treatment of proteins, for each sample, 500 ng of total peptides was separated and analyzed with a nano-UPLC (Vanquish neo) coupled to an Astral instrument (Thermo Scientific, Waltham, MA, USA) with a nano-electrospray ion source. Separation was performed using a reversed-phase column (EASY-Spray™ HPLC (150 μm × 15 cm), Thermo Scientific, Waltham, MA, USA). Mobile phases were H_2_O with 0.1% FA (phase A) and 80% ACN with 0.1% FA (phase B). Separation of the sample was executed with an 8 min gradient. Data-independent acquisition (DIA) was performed in profile and positive mode with an Orbitrap analyzer at a resolution of 240 K and an m/z range of 380–980 for MS1; for MS2, the m/z range was 150–2000. HCD was applied with a normalized collision energy (NCE) of 25% and an isolation window of 2 m/z.

### 2.7. DIA-NN Database Search

Vendor raw MS files were processed using DIA-NN software (1.8.1). MS spectral libraries were searched against their species-level UniProt FASTA database (uniprot_Mus musculus_10090_reviewe_2024.fasta), with Carbamidomethyl [C] as a fixed modification, and Oxidation (M) and Acetyl (Protein N-term) as variable modifications. Trypsin was used as a protease. A maximum of 2 missed cleavages was allowed. The false discovery rate (FDR) was set to 0.01 for both the PSM and peptide levels. Peptide identification was performed with an initial precursor mass deviation of up to 20 ppm and a fragment mass deviation of 20 ppm. All other parameters were retained as default. Missing values in the raw data were recoded (Missing Value Recoding) and imputed using the half-minimum method. No further data normalization was applied to the DIA-NN output matrix.

### 2.8. Bioinformatics Analysis

Bioinformatics analysis was performed using SIMCA software (V18.0.1, Sartorius Stedim Data Analytics AB, Umea, Sweden) for logarithmic and centralization processing of data, followed by principal component analysis (PCA) to visualize differentially expressed protein results in volcano plots. Differentially expressed proteins (DEPs) were screened using statistical methods, with the criteria set as a *p*-value < 0.05 (Student’s *t*-test or Chi-square test) and a fold change ≤ 0.83 or ≥1.2. In the statistical analysis of differentially expressed proteins, the Benjamini–Hochberg (BH) method was used for false discovery rate (FDR) correction. Raw *p*-values were converted to *q*-values (FDR values) after BH adjustment. Clusters of Orthologous Groups (COG) analysis was employed for homology classification of gene products. Subcellular localization analysis determined the specific intracellular localization of differentially expressed proteins. Gene mapping was conducted to various nodes in the Gene Ontology (GO) database of Mus musculus (mouse), followed by GO functional enrichment analysis (http://www.geneontology.org/ (accessed on 24 July 2025)). The Kyoto Encyclopedia of Genes and Genomes (KEGG) database was utilized to analyze significantly enriched metabolic pathways for differentially expressed proteins.

### 2.9. Measurement of Cellular Reactive Oxygen Species (ROS) Levels

Reactive oxygen species (ROS) levels in GC-1 cells were measured using a DCFH-DA assay kit (Beyotime, Shanghai, China) and flow cytometry (BD, Franklin Lakes, NJ, USA). Cells were seeded in 6-well plates at a density of 5 × 10^5^ cells per well and cultured for 24 h, followed by drug treatment for 24 h; the positive control group was treated for 1 h. Subsequently, cells were stained with 10 μmol/L DCFH-DA in the dark at 37 °C for 30 min, after which the DCFH-DA solution was removed by washing with serum-free medium. Cells were harvested and analyzed by flow cytometry.

### 2.10. Measurement of Total Glutathione and Lipid Peroxidation

GC-1 cells were seeded in 6-well plates at a density of 5 × 10^5^ cells per well and cultured for 24 h, followed by drug treatment for an additional 24 h. For the measurement of total glutathione (GSH) content, cells were collected according to the manufacturer’s instructions for the GSH assay kit (Beyotime, Shanghai, China). After adding the protein removal reagent, the samples were subjected to two rapid freeze–thaw cycles, incubated on ice, and centrifuged at 10,000× *g* for 10 min at 4 °C. The supernatant was collected, and the total GSH level was determined using a microplate reader. For the measurement of malondialdehyde (MDA) content, cells were lysed with the provided lysis buffer, and the lysates were centrifuged at 10,000× *g* for 10 min at 4 °C. The supernatant was then assayed according to the manufacturer’s protocol for the MDA assay kit (Beyotime, Shanghai, China). Briefly, the detection working solution was added to the samples, which were heated at 100 °C for 15 min. The MDA concentration was subsequently measured with a microplate reader.

### 2.11. Measurement of Fe^2+^ Content

GC-1 cells were seeded in 6-well plates at a density of 5 × 10^5^ cells per well and cultured for 24 h prior to drug treatment for an additional 24 h. For Fe^2+^ quantification, the Fe^2+^ assay kit (Beyotime, Shanghai, China) was used according to the manufacturer’s instructions. After treatment, cells were collected by centrifugation to obtain a cell pellet. The pellet was fully lysed with the provided lysis buffer, followed by centrifugation at 12,000 rpm for 5 min at 4 °C. The resulting supernatant was used as the test sample. A standard curve was prepared using 0–100 μM ferrous ion standard solutions. In a 96-well plate, control wells, standard wells, and sample wells were set up. To each well, 100 μL of standard solution or sample was added, followed by 5 μL of Decontamination Solution and 85 μL of Assay Buffer. After thorough mixing, the plate was incubated at 37 °C for 30 min. Absorbance was measured at 593 nm using a microplate reader, and Fe^2+^ concentrations were calculated based on the standard curve.

### 2.12. Quantitative RT-PCR Analysis

Total RNA was extracted from mouse testes using a commercial RNA extraction kit (Sicoge, Qinan, China) and quantified. RNase-free H_2_O was thawed at room temperature (15–25 °C) and immediately placed on ice. gDNA Eraser and 5 × qRT Mix were also prepared on ice. For reverse transcription, the mRNA reverse transcription reagents (Sparkjade, Qinan, China) were sequentially added to PCR tubes, mixed thoroughly by pipetting, and then incubated in a PCR thermal cycler at 50 °C for 15 min followed by 85 °C for 5 s. After cooling, the cDNA was stored for subsequent use. For quantitative PCR, reaction mixtures containing the cDNA template, primers (Table S1), and other qPCR reagents (Sparkjade, Qinan, China) were prepared in labeled 8-strip PCR tubes, mixed thoroughly, and loaded onto the instrument. The thermal cycling program was set as follows: initial denaturation at 94 °C for 15 s, followed by 40 cycles of 94 °C for 15 s, 60 °C for 20 s, and 72 °C for 25 s.

### 2.13. Western Blot

Polyacrylamide gels were prepared using a commercial PAGE gel kit (Epizyme, Shanghai, China). Protein samples (20 μg per lane) were separated by gel electrophoresis. Separated proteins were transferred onto a PVDF membrane (Millipore, Burlington, MA, USA) using a wet transfer method. The membrane was blocked with rapid blocking buffer (Epizyme, Shanghai, China) at room temperature for 20 min, followed by incubation with primary antibodies (Bioss, Beijing, China) at 4 °C overnight. After washing three times with TBST, the membrane was incubated with a secondary antibody (Proteintech, Wuhan, China) at room temperature for 1 h, followed by three additional TBST washes. Protein bands were visualized by adding ECL chemiluminescence substrate (Yeasen, Shanghai, China) and imaged. Band intensity was quantified using ImageJ (v1.8.0) software. Antibody information is shown in Table S2.

### 2.14. HE Staining

Mouse testicular tissues were fixed in 4% paraformaldehyde, dehydrated through a graded ethanol series and xylene, and embedded in paraffin for sectioning. After deparaffinization, the sections were stained with Hematoxylin and Eosin staining solution (Solarbio, Beijing, China) and mounted with neutral balsam. The morphological structure of the testicular tissue and the number of spermatogonia within the seminiferous tubules were examined under an optical microscope.

### 2.15. Immunofluorescence (IF)

Following deparaffinization, paraffin-embedded sections of mouse testicular tissue were subjected to antigen retrieval. The sections were then incubated with 5% BSA blocking solution at room temperature for 1 h. The primary antibody was applied, and the sections were incubated overnight at 4 °C in a humidified chamber. After three washes with PBST, the sections were incubated with a fluorescently labeled secondary antibody (Abcam, Cambridge, UK) at a 1:500 dilution for 1 h at room temperature in the dark. After three washes with PBST, the sections were mounted in Prolong Gold antifade reagent containing DAPI (Life Technologies, Grand Island, NY, USA). The samples were observed under a fluorescence microscope. Antibody information is shown in Table S2.

### 2.16. Statistical Analysis

All statistical analyses were carried out using GraphPad Prism 8.0 or SPSS 25.0. Data normality was assessed using the Shapiro–Wilk test. For data following a normal distribution, homogeneity of variances was further evaluated using Levene’s test. When both assumptions of normality and homogeneity of variances were met, comparisons between two groups were performed using Student’s *t*-test, and comparisons among multiple groups were performed using one-way analysis of variance (ANOVA), with statistical significance defined as *p* < 0.05. For data that did not meet the assumptions of normality or homogeneity of variances, the Mann–Whitney U test was used for two-group comparisons, and the Kruskal–Wallis H test followed by Bonferroni correction was used for multiple-group comparisons.

## 3. Results

### 3.1. Effects of Curcumin on Proliferation and Apoptosis of TP-Damaged GC-1 Cells

**Figure 1:**
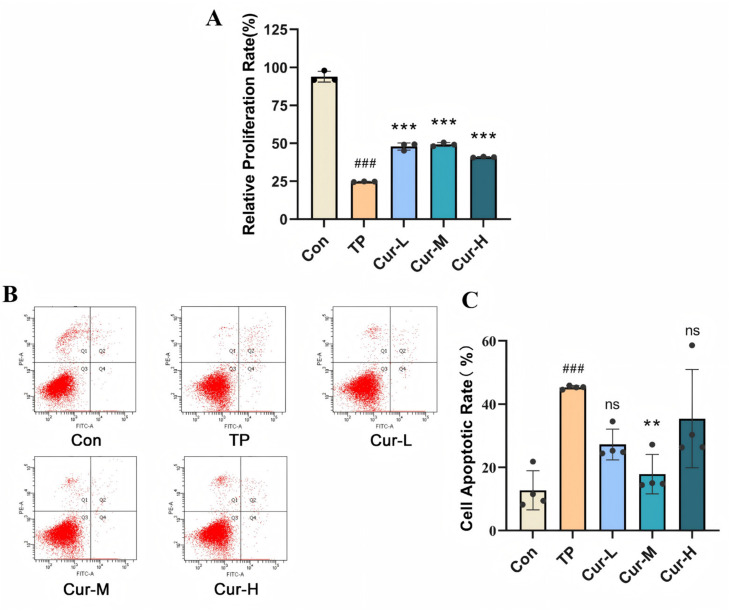
Proliferation and apoptotic status of GC-1 cells. (**A**) Results of cell proliferation rate determined by the CCK-8 assay (*n* = 3). (**B**) Flow cytometry analysis of cell apoptosis in each group. (**C**) Statistical results of the apoptosis rate (*n* = 4). Significance levels are denoted as follows: ### *p* < 0.001 compared with the Con group; ** *p* < 0.01, *** *p* < 0.001 compared with the TP group. ns: no significant differences.

Cell proliferation was evaluated using the CCK-8 method, which detects mitochondrial dehydrogenase activity, and revealed that compared with the Con group, the TP group exhibited a significant decrease in cell proliferation rate (*p* < 0.001). Following curcumin intervention, the proliferation rates in all curcumin-treated groups were significantly increased relative to the TP group (*p* < 0.001) (Figure 1A). Apoptosis analysis (Figure 1B) showed that compared with the Con group, the TP group displayed a highly significant increase in apoptosis rate (*p* < 0.001). After curcumin intervention, the Cur-M group showed a marked reduction in apoptosis rate (*p* < 0.01). These results indicate that TP treatment reduces GC-1 cell proliferation and increases apoptosis, while curcumin intervention significantly attenuates TP-induced apoptosis and enhances proliferation. Among the tested concentrations, the 4 µM curcumin group (Cur-M) demonstrated the most pronounced restoration of proliferation and apoptosis levels.

### 3.2. Identification of Differentially Expressed Proteins in GC-1 Cells

**Figure 2:**
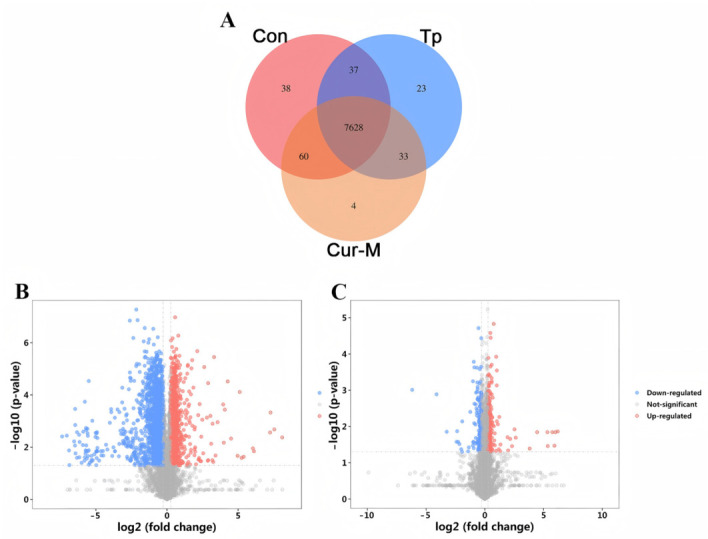
Inter-group reproducibility Venn diagram and differentially expressed protein volcano plots (*n* = 3). (**A**) Venn diagram illustrating the reproducibility analysis of protein identification across sample groups. A total of 7628 reproducible proteins were identified. Specifically, thirty-eight proteins were uniquely identified in the Con group, twenty-three proteins in the TP group, and four proteins in the Cur-M group. (**B**) Volcano plot of differentially expressed proteins for the comparison between the Con and TP groups. (**C**) Volcano plot of differentially expressed proteins for the comparison between the TP and Cur-M groups.

Based on the aforementioned experimental results, we employed the Con group, TP group, and Cur-M group for differential proteomic analysis. Each sample was analyzed using DIA mode for protein identification and quantification, retaining proteins with at least one unique peptide. Differentially expressed proteins (DEPs) were screened using statistical methods, with the criteria set as a *p*-value < 0.05 (Student’s *t*-test or Chi-square test) and a fold change ≤ 0.83 or ≥1.2. The reproducibility analysis between group samples is shown in Figure 2A, where 7628 reproducible proteins were identified. In the comparison between the Con group and the TP group, a total of 3129 DEPs were found, comprising 1277 upregulated and 1852 downregulated proteins (Figure 2B, Table S3). Between the TP group and the Cur-M group, a total of 378 DEPs were identified, including 202 upregulated and 176 downregulated proteins (Figure 2C, Table S4).

### 3.3. Results of Bioinformatics Analysis of Differentially Expressed Proteins

**Figure 3:**
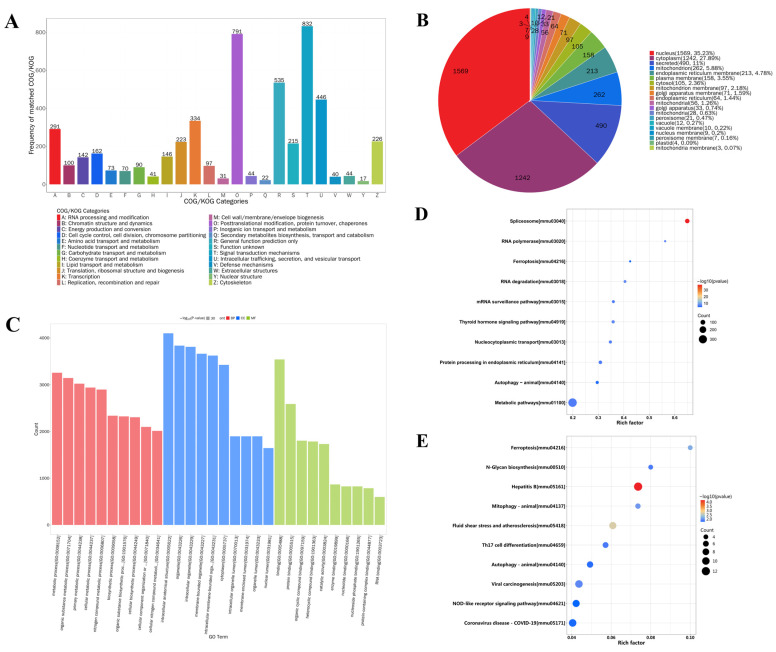
Results of bioinformatics analysis (*n* = 3). (**A**) Histogram of COG analysis for differentially expressed proteins: The horizontal axis represents the functional categories of COG classification, and the vertical axis indicates the frequency of COG assignments. (**B**) Pie chart illustrating the subcellular localization analysis of differentially expressed proteins. (**C**) Bar chart of GO enrichment analysis for the comparison Con vs. TP vs. Cur-M. (**D**) Bubble plot of KEGG pathway enrichment analysis for the comparison Con vs. TP. (**E**) Bubble plot of KEGG pathway enrichment analysis for the comparison TP vs. Cur-M.

COG analysis revealed that the differentially expressed proteins (DEPs) identified in the comparisons Con vs. TP vs. Cur-M were primarily annotated to functions such as Signal transduction mechanisms, Posttranslational modification, protein turnover, chaperones, General function prediction only, Intracellular trafficking, secretion, and vesicular transport, Transcription, and RNA processing and modification (Figure 3A, Table S5). Subcellular localization analysis indicated that the majority of DEPs from the Con vs. TP vs. Cur-M comparisons were distributed in the nucleus, accounting for approximately 35% of the total, with 28% located in the Cytoplasm (Figure 3B, Table S6).

**Figure 4:**
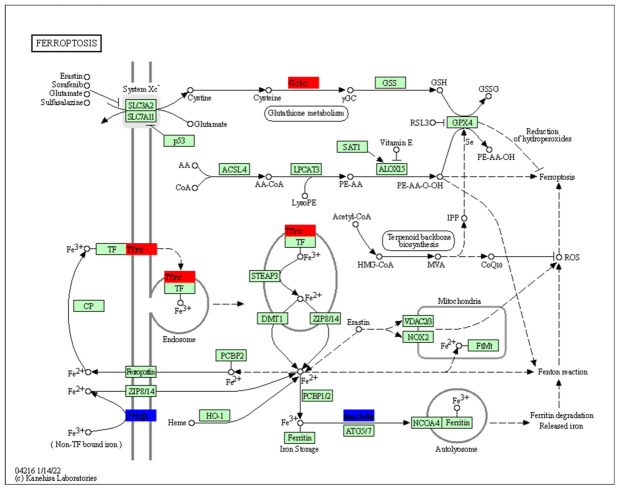
Ferroptosis pathway map [23] for the TP vs. Cur-M group. Using the TP group as a control, red indicates upregulation of protein expression in the Cur-M group, and blue indicates downregulation. In the Cur-M group, TFRC and MAP1LC3A were upregulated, whereas PRNP and GCLC were downregulated.

GO enrichment analysis demonstrated that the DEPs were predominantly enriched in biological processes, including metabolic process, organic substance metabolic process, and primary metabolic process; cellular components such as intracellular anatomical structure, Organelle, and intracellular Organelle; and molecular functions including Binding, protein binding, and organic cyclic compound binding (Figure 3C, Table S7). KEGG pathway enrichment analysis identified that DEPs from the Con vs. TP comparison were mainly enriched in pathways including Spliceosome, RNA polymerase, Ferroptosis, RNA degradation, mRNA surveillance pathway, Thyroid hormone signaling pathway, Nucleocytoplasmic transport, Protein processing in endoplasmic reticulum, Autophagy-animal, and Metabolic pathways (Figure 3D, Table S8). DEPs from the TP vs. Cur-M comparison were primarily enriched in pathways such as Ferroptosis, N-Glycan biosynthesis, Hepatitis B, Mitophagy-animal, Fluid shear stress and atherosclerosis, Th17 cell differentiation, Autophagy-animal, Viral carcinogenesis, and NOD-like receptor signaling pathway (Figure 3E, Table S9). The pathways commonly enriched by DEPs across all three comparisons (Con vs. TP vs. Cur-M) were Ferroptosis and Autophagy-animal. Notably, within the ferroptosis pathway of the TP vs. Cur-M comparison, the expression of Transferrin Receptor Protein 1 (TFRC) and Microtubule-associated protein 1 light chain 3 alpha (MAP1LC3A) was upregulated. In contrast, the expression of Prion Protein (PRNP) and Glutamate–Cysteine Ligase Catalytic Subunit (GCLC) was downregulated (Figure 4, Table S10) [23]. The bioinformatics analysis indicates that the protective effect of curcumin against TP-induced GC-1 cell injury is highly associated with the ferroptosis pathway.

### 3.4. Curcumin Inhibits Ferroptosis in GC-1 Cells and Protects Against TP-Induced Damage

**Figure 5:**
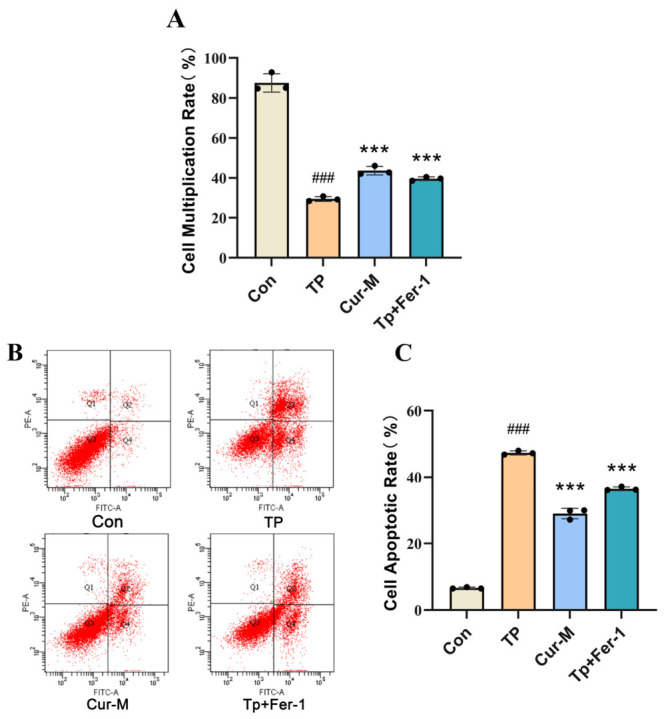
Analysis of cell proliferation rate and apoptosis rate (*n* = 3). (**A**) CCK-8 cell proliferation results for each group. (**B**) Flow cytometry analysis plots of apoptosis rate for each group. (**C**) Statistical graph of apoptosis rates for each group. Significance levels are denoted as follows: ### *p* < 0.001 compared with the Con group; *** *p* < 0.001 compared with the TP group.

To verify the effect of curcumin on ferroptosis in TP-damaged GC-1 cells, we performed CCK-8 cell proliferation assays. Compared with the TP group, the Cur-M group showed a highly significant increase in cell proliferation rate (*p* < 0.001), and the TP+Fer-1 group also showed a highly significant increase (*p* < 0.001) (Figure 5A). Apoptosis assays revealed that the apoptosis rate in the Cur-M group was significantly lower than that in the TP group (*p* < 0.001). The TP+Fer-1 group also showed a highly significant reduction (*p* < 0.001) (Figure 5B). This indicates that both curcumin and the ferroptosis inhibitor effectively reduced TP-induced apoptosis and increased GC-1 cell proliferation.

**Figure 6:**
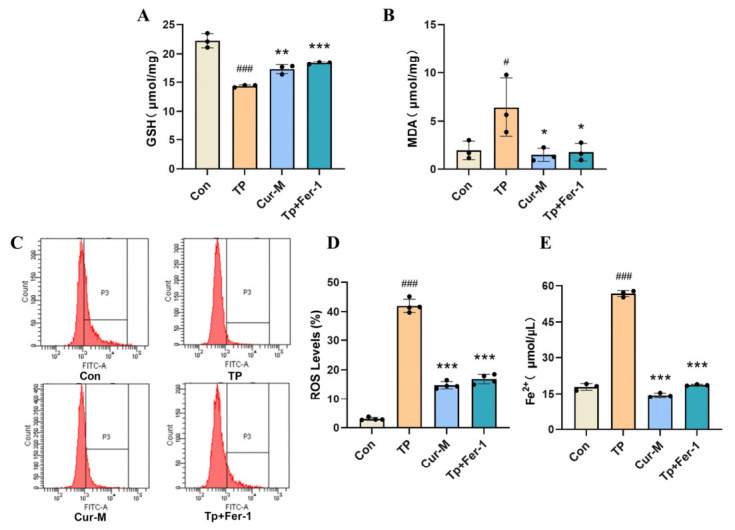
Levels of total glutathione, MDA, ROS, and Fe^2+^ in cells (*n* = 3). (**A**) Cellular total glutathione (GSH) levels. (**B**) Cellular lipid peroxidation (MDA) levels. (**C**) Flow cytometry analysis plots of cellular ROS levels. (**D**) Statistical graph of cellular ROS levels. (**E**) Intracellular Fe^2+^ content. Significance levels are denoted as follows: # *p* < 0.05, ### *p* < 0.001 compared with the Con group; * *p* < 0.05, ** *p* < 0.01, *** *p* < 0.001 compared with the TP group.

Total glutathione level detection showed that GSH in the TP group was significantly lower than in the Con group (*p* < 0.001). Compared with the TP group, GSH in the Cur-M group was significantly increased (*p* < 0.01). In the TP+Fer-1 group, it was highly significantly increased (*p* < 0.001) (Figure 6A). Detection of MDA, ROS, and Fe^2+^ levels showed that the concentrations of MDA, ROS, and Fe^2+^ in both the Cur-M and TP+Fer-1 groups were highly significantly lower than those in the TP group (*p* < 0.001) (Figure 6B–E). This indicates that both curcumin and the ferroptosis inhibitor significantly increased intracellular GSH content, reduced reactive oxygen species and Fe^2+^ levels, lowered lipid peroxidation, and inhibited ferroptosis in GC-1 cells.

### 3.5. Curcumin Regulates the Expression of Nrf2/Gclc/Map1lc3a/Tfrc/Dmt1 in GC-1 Cells to Inhibit TP-Induced Ferroptosis

**Figure 7:**
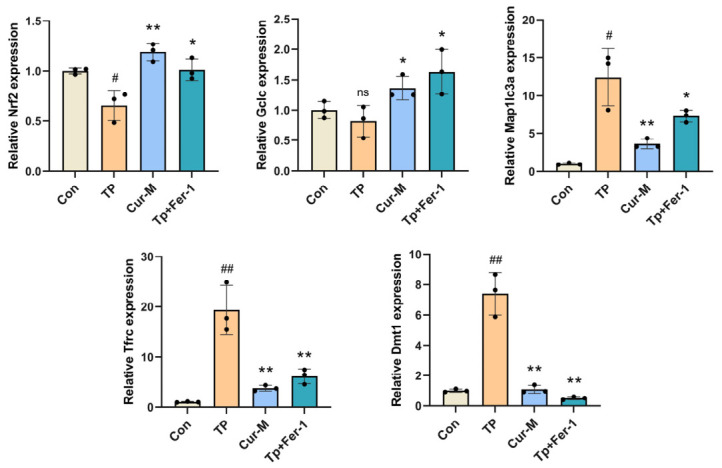
Relative mRNA expression levels of *Nrf2*, *Gclc*, *Map1lc3a*, *Tfrc*, and *Dmt1* in GC-1 cells (*n* = 3). Significance levels are denoted as follows: # *p* < 0.05, ## *p* < 0.01 compared with the Con group; * *p* < 0.05, ** *p* < 0.01 compared with the TP group. ns: no significant differences.

To verify the molecular regulatory role of curcumin on ferroptosis in TP-damaged GC-1 cells, we performed qPCR and Western blot (WB) experiments to detect the expression of ferroptosis pathway-related molecules: *Nrf2*, *Gclc*, *Map1lc3a*, *Tfrc*, and *Dmt1*. qRT-PCR results showed that compared with the Con group, the relative mRNA expression of *Nrf2* in GC-1 cells of the TP group was significantly decreased (*p* < 0.05). In contrast, the relative mRNA expression of *Map1lc3a, Tfrc*, and *Dmt1* was significantly increased (*p* < 0.05). Compared with the TP group, the Cur-M and TP+Fer-1 groups showed significantly increased relative mRNA expression of *Nrf2* and *Gclc* (*p* < 0.05) and significantly decreased relative mRNA expression of *Map1lc3a*, *Tfrc*, and *Dmt1* (*p* < 0.05) (Figure 7).

**Figure 8:**
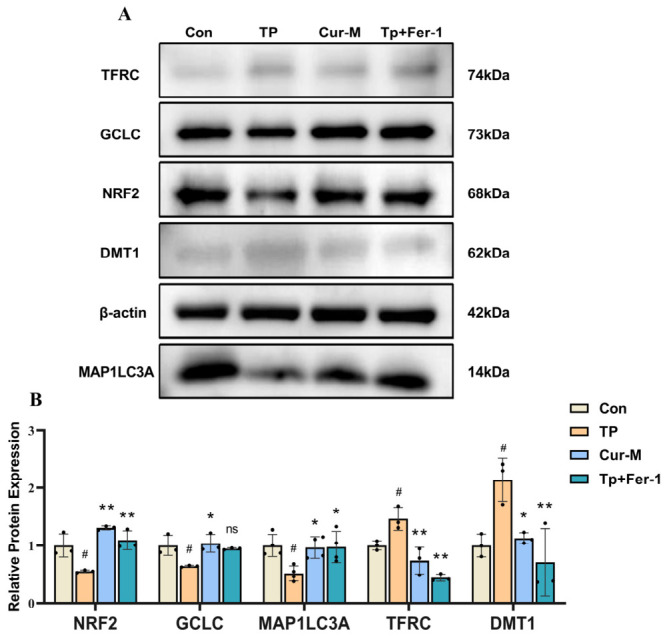
Western blot (WB) detection results in GC-1 cells (*n* = 3). (**A**) Protein bands of NRF2, GCLC, MAP1LC3A, TFRC, and DMT1 in GC-1 cells (using β-actin as control). (**B**) Statistical graph of band grayscale values. Significance levels are denoted as follows: # *p* < 0.05 compared with the Con group; * *p* < 0.05, ** *p* < 0.01 compared with the TP group. ns: no significant differences.

WB results showed that compared with the Con group, the protein expression levels of NRF2, GCLC, and MAP1LC3A in GC-1 cells of the TP group were significantly decreased (*p* < 0.05). In contrast, the expression levels of TFRC and DMT1 were significantly increased (*p* < 0.05). Compared with the TP group, the Cur-M and TP+Fer-1 groups showed significantly increased protein expression levels of NRF2, GCLC, and MAP1LC3A (*p* < 0.05) and significantly decreased protein expression levels of TFRC and DMT1 (*p* < 0.05) (Figure 8). These results indicate that curcumin can ameliorate TP-induced ferroptosis damage in GC-1 cells by regulating the expression of *Nrf2*/*Gclc*/*Map1lc3a*/*Tfrc*/*Dmt1*.

### 3.6. Curcumin Regulates the Expression of Nrf2/Gclc/Map1lc3a/Tfrc/Dmt1 in Testes and Inhibits TP-Induced Ferroptosis

**Figure 9:**
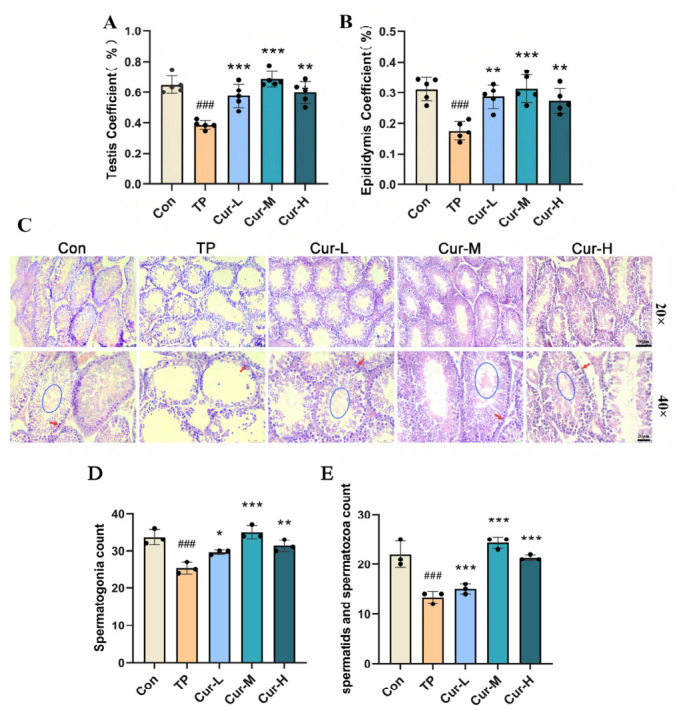
Testicular and epididymal coefficients and histological structure in mice (*n* = 5). (**A**) Testicular coefficient in mice. (**B**) Epididymal coefficient in mice. (**C**) HE-stained sections of testicular tissue. Red arrows indicate spermatogonia within the testis; blue circles highlight mature sperm within the seminiferous tubules. (**D**) Statistical analysis of spermatogonia numbers in HE-stained sections of testicular tissue. (**E**) Statistical analysis of spermatid and spermatozoa numbers in HE-stained sections of testicular tissue. Significance levels are denoted as follows: ### *p* < 0.001 compared with the Con group; * *p* < 0.05, ** *p* < 0.01, *** *p* < 0.001 compared with the TP group.

We performed an in vivo experiment to further verify the effect of curcumin on ferroptosis in TP-damaged mouse spermatogonia. Statistical analysis of testes and epididymides from each group showed that TP reduced the mass of testes and epididymides in mice. At the same time, curcumin significantly improved the mass of both testes and epididymides (*p* < 0.01) (Figure 9A,B). Observation of HE-stained sections of mouse testicular tissue (Figure 9C) revealed that compared with the Con group, the TP group exhibited a significant decrease in the number of spermatogonia and mature sperm within the seminiferous tubules, with even shedding of various spermatogenic cells and increased inter-tubular spaces. After curcumin intervention, all treated groups showed a significant increase in the number of spermatogonia and sperm within the seminiferous tubules compared with the TP group, and the tubules were arranged more closely together. Among them, the Cur-M group showed numbers of spermatogonia and mature sperm similar to those of the Con group, indicating the most pronounced therapeutic effect of curcumin.

**Figure 10:**
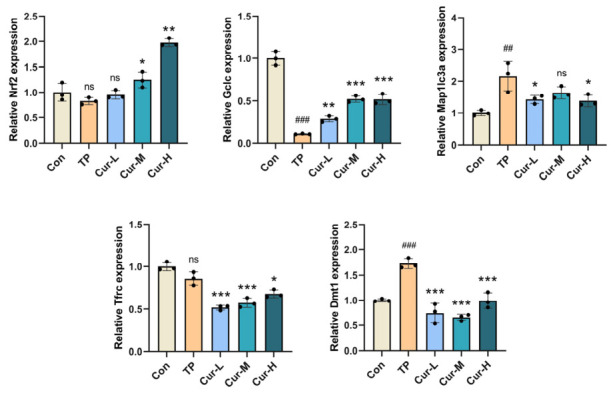
Relative mRNA expression levels of *Nrf2*, *Gclc*, *Map1lc3a*, *Tfrc*, and *Dmt1* in mouse testes (*n* = 3). Significance levels are denoted as follows: ## *p* < 0.01, ### *p* < 0.001 compared with the Con group; * *p* < 0.05, ** *p* < 0.01, and *** *p* < 0.001 compared with the TP group. ns: no significant differences.

**Figure 11:**
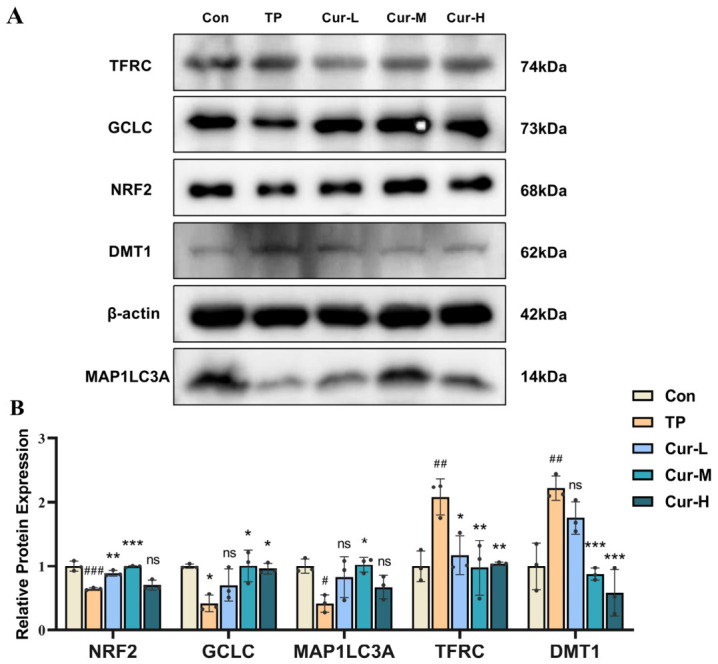
Western blot (WB) detection results in mouse testes (*n* = 3). (**A**) Protein bands of NRF2, GCLC, MAP1LC3A, TFRC, and DMT1 in mouse testes (with β-actin as the loading control). (**B**) Statistical analysis of band grayscale values. Significance levels are denoted as follows: # *p* < 0.05, ## *p* < 0.01, and ### *p* < 0.001 compared with the Con group; * *p* < 0.05, ** *p* < 0.01, and *** *p* < 0.001 compared with the TP group. ns: no significant differences.

**Figure 12:**
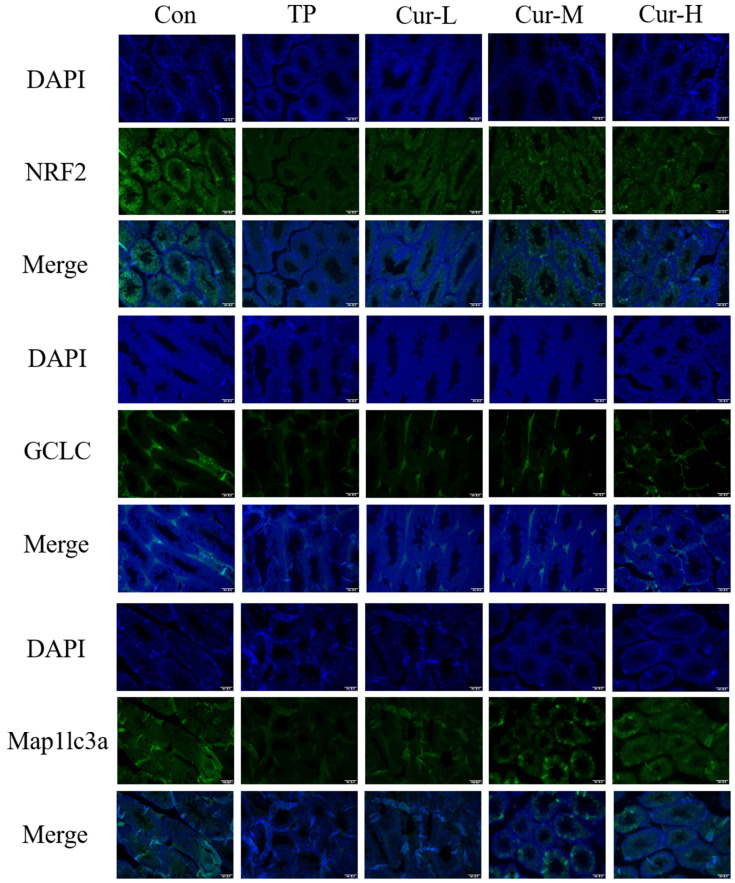

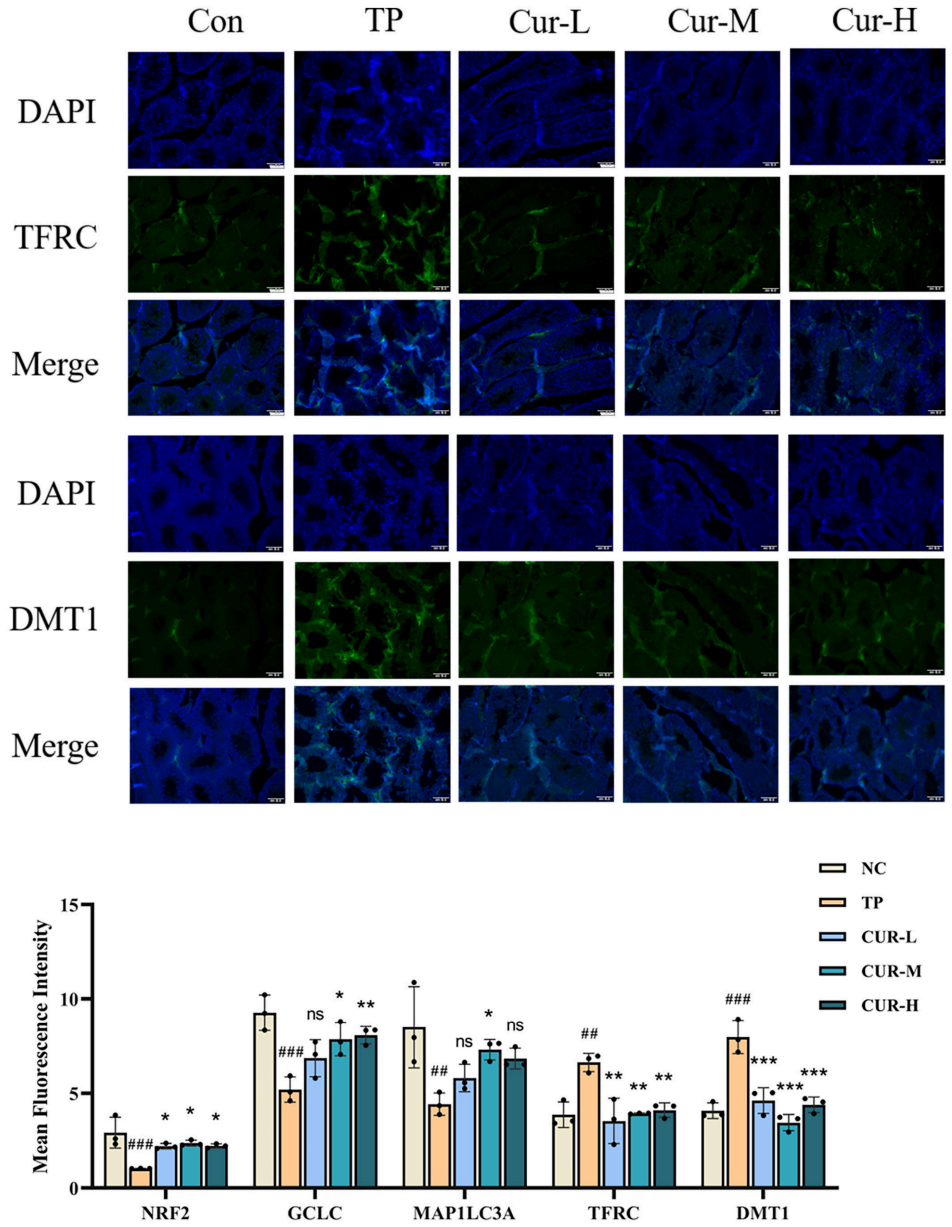
Immunofluorescence and fluorescence intensity statistics in mouse testis (*n* = 3). Staining was performed using antibodies against Nrf2, GCLC, MAP1LC3A, TFRC, and DMT1, with DAPI for nuclear counterstaining. Images were captured under a fluorescence microscope, and fluorescence intensity was quantified using ImageJ software. Scale bar = 50 μm. Significance levels are denoted as follows: ## *p* < 0.01, ### *p* < 0.001 compared with the Con group; * *p* < 0.05, ** *p* < 0.01, and *** *p* < 0.001 compared with the TP group. ns: no significant differences.

Through qRT-PCR validation of relative mRNA levels in mouse testicular tissue and WB experiments to verify protein levels, results similar to those in GC-1 cells were observed (Figure 10 and Figure 11). Compared with the Con group, the TP group showed significantly decreased mRNA and protein expression levels of *Nrf2*, *Gclc*, and *Map1lc3a* in testicular tissue, along with significantly increased expression of *Tfrc* and *Dmt1*. Following curcumin intervention, each treated group exhibited significantly upregulated expression of *Nrf2*, *Gclc*, and *Map1lc3a* and downregulated expression of *Tfrc* and *Dmt1* at both the mRNA and protein levels. Immunofluorescence experiments (Figure 12) revealed that NRF2 and MAP1LC3A are mainly distributed in spermatogonia, spermatids, and Sertoli cells. In contrast, GCLC, TFRC, and DMT1 were mainly distributed in spermatogenic cells and Leydig cells. It should be noted that cell types were distinguished based on morphological criteria.

## 4. Discussion

Triptolide is widely recognized as a promising clinical antitumor agent, and TP and its derivatives have been used to treat cancers such as breast, ovarian, and pancreatic cancers [24,25,26]. However, the significant toxicity of TP to the reproductive system limits its clinical application. TP can markedly reduce sperm motility in mice and disrupt the integrity of the blood–testis barrier by down-regulating tight-junction (TJ) protein expression in the testes. After normal female rats mated with male rats administered TP, the pregnancy rate decreased by 100% [27,28]. In testicular tissues in mice treated with TP, spermatogenic cells exhibit mitochondrial-mediated apoptosis; furthermore, TP-induced disturbances in lipid and energy metabolism in the testes may contribute to impaired spermatogenesis [29,30]. Curcumin can enhance the activities of SOD, CAT, and GSH-Px, elevate Nrf2 levels, facilitate Nrf2 translocation into the nucleus to regulate the expression of HO-1 and GCLC, thereby eliminating ROS and reducing intracellular reactive oxygen species concentrations [31]. Research has demonstrated that curcumin possesses potential protective effects against male reproductive injury [12,13,32]. In this study, TP exposure resulted in significant physiological damage to mouse testes, elevated oxidative stress in mouse spermatogonia, and increased cell apoptosis. Curcumin significantly reduced apoptosis, ROS levels, lipid peroxidation, and Fe^2+^ content in TP-induced GC-1 cells, while elevating intracellular GSH levels. These results are consistent with those of the above-cited studies.

DIA can significantly improve data completeness and enhance detection sensitivity and depth. In this study, DIA proteomics revealed 3129 differentially expressed proteins between the control group and the TP group and 378 differentially expressed proteins between the TP group and the Cur-M group. KEGG pathway analysis indicated that these differentially expressed proteins were all associated with the ferroptosis pathway. GO analysis showed that the functions of these proteins were enriched in metabolic processes and binding activities, which are related to iron/lipid/glutathione metabolism and protein binding events in ferroptosis. Subcellular localization was primarily enriched in the cytoplasm and nucleus, consistent with the cytoplasmic sites of iron metabolism, Nrf2 nuclear translocation, and the nuclear core of transcriptional regulation. Therefore, the protective effect of curcumin against TP-induced GC1 cell injury is strongly correlated with the ferroptosis pathway.

Ferroptosis is triggered by the inactivation of glutathione (GSH)-dependent antioxidant defenses in cells, leading to the accumulation of toxic lipid ROS (L-ROS) and the induction of lipid peroxidation [33,34]. Ferroptosis involves abnormalities in iron metabolism, lipid metabolism, and glutathione metabolism. In iron metabolism, iron uptake, storage, and release are critical for ferroptosis; excess ferrous ions catalyze the generation of lipid peroxides via the Fenton reaction, a key chemical reaction driving ferroptosis [35,36]. Ferritin and transferrin binding to iron are also particularly important in the ferroptosis process [37,38]. The RNA-binding protein NKAP can inhibit ferroptosis by binding to m6A-modified cystine transporter SLC7A11 mRNA and promoting its up-regulation [39]; these proteins can regulate ferroptosis through their binding functions. Under conditions such as oxidative stress, Nrf2 translocates into the nucleus, initiating the transcription of downstream antioxidant and cytoprotective genes (e.g., SLC7A11, GPX4, FTH1). After translation, the resulting proteins return to the cytoplasm and organelles to perform their functions, thereby maintaining iron homeostasis and suppressing ferroptosis [40,41]. Several studies have already discovered that TP can induce ferroptosis. For example, TP can inactivate the source of glutathione (GSH) biosynthesis, thereby activating ferroptosis in glioblastoma cells by blocking the neutralization of intracellular lipid peroxides [42]. In the reproductive system, TP can promote GPX4 ubiquitination and degradation, stimulating ferroptosis in spermatogenic cells and resulting in impaired spermatogenesis [10]. Concurrently, numerous studies have shown that curcumin can inhibit ferroptosis in various tissues. Curcumin significantly elevates the expression levels of SLC7A11 and GPX4 while suppressing ACSL4 and TFRC expression, exerting a protective effect against ferroptosis in mice with periodontitis [43]. Curcumin can promote Nrf2 nuclear translocation, increase the expression of oxidative scavenging factors such as HO-1, and inhibit glucose-induced ferroptosis in cardiomyocytes [44]. Zearalenone-induced ferroptosis leads to obvious loss of mitochondrial cristae and membrane collapse, which can be markedly alleviated by curcumin intervention [45]. Moreover, curcumin can attenuate dibutyl phthalate-induced ferroptosis by upregulating SP1 and PRDX6, thereby protecting testicular tissue from ferroptotic damage [46].

Nrf2 maintains cellular redox homeostasis under oxidative stress, exerting antioxidant, anti-inflammatory, and anti-apoptotic effects, and is a key regulator of cellular homeostasis [47,48,49]. *Nrf2* and its coordinated genes are widely expressed in the testes, where they play crucial roles in regulating the blood–testis barrier and defending against testicular toxic injury [50,51]. Studies indicate that testicular oxidative stress leads to down-regulation of *Nrf2* gene expression, accompanied by increased lipid peroxidation, germ cell death, and decreased antioxidant capacity in the testes [50,52]. Cadmium and molybdenum jointly induce apoptosis and ferroptosis in duck testes by inhibiting the *Nrf2* pathway [53]. In some oligospermic patients, Nrf2 deficiency leads to down-regulation of certain ferroptosis-related genes, promoting ferroptosis and ultimately contributing to oligospermia [54]. GCLC is the rate-limiting enzyme in glutathione (GSH) synthesis and is involved in regulating intracellular glutamate homeostasis. In the initial step of GSH production, the conjugation of cysteine and glutamate is catalyzed by GCLC. By synthesizing GSH and scavenging lipid peroxides, GCLC serves as a major protective mechanism against ferroptosis [55,56]. Research on T cells has shown that upregulation of GCLC prevents ferroptosis induced by cystine deprivation, thereby maintaining the antitumor function of T cells [57]. In studies on diabetic rats, treatment with CeO2 nanoparticles upregulated GCLC expression, enhanced antioxidant capacity, and conferred protection against testicular injury in diabetic rats [58]. Furthermore, *Gclc* is one of the target genes regulated by *Nrf2*; *Nrf2* activation can upregulate *Gclc* expression, increase GSH synthesis, enhance cellular antioxidant capacity, and thereby inhibit ferroptosis [59,60].

TFRC (Transferrin Receptor) is a key transporter for cellular iron uptake. Up-regulation of TFRC predicts increased intracellular iron, promoting lipid peroxidation; down-regulation of TFRC directly reduces iron uptake across the cell membrane, alleviating lipid peroxidation damage and decreasing ferroptosis [61,62]. Studies have shown that overexpression of TFRC in porcine testes reduces testicular cell viability, increases cytotoxicity, exacerbates oxidative stress injury, diminishes mitochondrial activity, and promotes ferroptosis [63]. Moreover, pharmacological reduction or knockdown of TFRC levels can attenuate ferroptosis and significantly ameliorate ferroptosis-induced damage to the blood–testis barrier [64,65]. DMT1 (Divalent Metal Transporter 1) is a critical iron transporter on the cell membrane and endoplasmic reticulum. Fe^3+^ is transported in serum by transferrin (TF), then binds to TFRC on the cell membrane and is internalized. Fe^3+^ is reduced to Fe^2+^, which is subsequently released into the cytoplasm by DMT1 [40,66]. In an intracerebral hemorrhage (ICH) model, promoting the degradation of DMT1 significantly reduced tissue iron content, inhibited ferroptosis, and improved neurological recovery [67]. In testicular research, both TFRC and DMT1 mRNA and protein expression were increased in testicular tissues of Nrf2-knockout mice, accompanied by decreased sperm concentration and motility and significantly reduced fertility, indicating that elevated DMT1 levels may cause testicular damage [54]. In the present study, it was found that in the TP-induced GC1 cell injury model and testicular injury mouse model, the protein expression of NRF2 and GCLC was significantly downregulated, while the expression of TFRC and DMT1 was notably upregulated. Following curcumin intervention, the expression levels of NRF2 and GCLC were markedly increased in both GC1 cells and testicular tissue, whereas the expression of TFRC and DMT1 was significantly decreased. Additionally, curcumin or Ferrostatin-1 treatment reduced ROS, Fe^2+^, and lipid peroxidation levels, and elevated GSH levels in GC-1 cells, which aligns with the regulatory outcomes of these proteins reported in previous studies. These findings suggest that curcumin effectively regulated ferroptosis-related pathways in both TP-induced GC-1 cells and mouse testes, thereby conferring a protective effect against GC-1 cell and testicular injury.

To date, there is no definitive literature confirming that Map1lc3a directly regulates ferroptosis; most research focuses on its role as an autophagy marker. Generally, upregulation of Map1lc3a may lead to increased intracellular free iron and promote ferroptosis [68]. However, within specific cytoprotective mechanisms, if accompanied by significant up-regulation of anti-ferroptosis regulatory molecules, up-regulation of Map1lc3a may instead become part of the cellular self-protection strategy, and the overall outcome may still suppress ferroptosis [69]. Studies have found that in Atg5-mutant mice, LC3A expression in the testes was significantly reduced, testicular autophagy activity was lowered, and the mice exhibited substantially decreased fertility, with sperm count and motility also significantly lower than in the control group [70]. In this study, it was observed that in the TP-induced mouse spermatogonial injury model, Map1lc3a mRNA expression levels were markedly increased, while after intervention with curcumin and a ferroptosis inhibitor, its mRNA levels decreased significantly. However, Map1lc3a protein expression showed the opposite trend. This may be because MAP1LC3A must undergo a series of post-translational processing events to gain function, and these modifications are what change the protein’s detectability. Upon synthesis, the MAP1LC3A precursor protein (pro-LC3A) is rapidly cleaved at its C-terminus to produce the lipidated form LC3-II [71]. This lipidation changes the physicochemical properties of LC3, increasing its stability or its affinity for membrane structures [72]. Therefore, even if transcription of the MAP1LC3A gene is downregulated (reduced mRNA), activation of autophagy can still promote the rapid conversion of pro-LC3A into the stable, membrane-bound LC3-II form, resulting in a stronger total LC3 or LC3-II signal on Western blots, interpreted as elevated protein levels. Moreover, interaction of LC3 with p62/SQSTM1 can promote the formation of protein aggregates, which may be relatively stable and resistant to rapid degradation, leading to higher signals on Western blots [73]. Under steady-state conditions, mRNA levels partly explain protein levels; however, the regulation of translational and degradation processes largely dictates the final protein abundance [74]. Moreover, miRNA-mediated translational inhibition hinders ribosome loading and promotes mRNA deadenylation, leading to reduced translation efficiency [71,75]. When autophagic flux is blocked or LC3A is excessively integrated into autophagosomes and degraded, a decrease in total protein levels may occur [76,77]. The present data do not allow for the complete exclusion of other possibilities; therefore, future research is needed to further investigate the specific expression mechanisms of Map1lc3a and whether there is a ferroptosis–autophagy interplay influencing the protective effect of curcumin against TP-induced injury in mouse spermatogonia.

A sophisticated and intricate signaling network exists between ferroptosis and autophagy, two forms of regulated cell death, in which autophagy acts as a key upstream regulator through multiple selective degradation pathways. The most direct manifestation of this interplay is ferritinophagy—the NCOA4-mediated selective autophagic degradation of ferritin. This process liberates substantial amounts of labile iron, which, in turn, drives lipid peroxidation via the Fenton reaction, ultimately triggering ferroptosis [78,79]. In addition to ferritinophagy, cells employ various other forms of selective autophagy. For example, lipophagy degrades lipid droplets, thereby providing substrates for lipid peroxidation. Meanwhile, chaperone-mediated autophagy directly degrades GPX4, a key inhibitor of ferroptosis, thus driving ferroptosis through an alternative pathway [80]. Furthermore, the autophagy-related protein p62/SQSTM1 links autophagy to ferroptosis by modulating the KEAP1–NRF2 antioxidant signaling axis. On one hand, it participates in the recognition and clearance of autophagic substrates; on the other hand, it enhances cellular antioxidant defenses through NRF2 activation, thereby counteracting ferroptosis and establishing a finely tuned feedback regulatory mechanism [81,82]. Other autophagy regulators, such as BECN1, AMPK, and mTOR, also play critical roles in cell fate determination. Together, they constitute a central regulatory network that integrates cellular metabolic status, redox balance, and degradation signals [83,84].

In most cases, the quantitative proteomics data obtained by mass spectrometry in this study showed good consistency with the Western blot validation results. However, TFRC and MAP1LC3A exhibited some discordance between the two datasets, which warrants further discussion. TFRC is a transmembrane protein whose function is subject to fine regulation by post-translational modifications (PTMs) and intracellular trafficking. TFRC can undergo multiple PTMs, including N-glycosylation, O-glycosylation, phosphorylation, and palmitoylation [85]. These modifications may alter its antigenic epitope and affect recognition efficiency by the Western blot antibody. In addition, differences in lysis conditions between Western blot and mass spectrometry sample preparation may affect the extraction efficiency of transmembrane proteins, which could be an important factor contributing to the discordant results obtained by the two methods. The MAP1LC3 family comprises three isoforms (LC3A, LC3B, and LC3C) that exhibit distinct subcellular distribution dynamics and expression profiles [86]. The discrepancy between mass spectrometry data (based on quantification of characteristic peptides) and Western blot data (based on antibody recognition) in LC3A detection may be attributed to antibody cross-reactivity and differential expression of LC3 isoforms.

This study investigated the effects of curcumin on mouse testes and spermatogonia following TP intervention. However, whether curcumin improves mouse sperm quality remains to be validated through experimental assessments of sperm morphology and motility. While the mouse model recapitulates the key molecular changes observed in GC-1 cells, it also provides essential physiological validation: histopathological evidence of preserved spermatogenesis and systemic safety of curcumin. However, our in vivo analyses were performed on whole-testis homogenates, which average signals from all testicular cell types. Therefore, we cannot determine whether curcumin acts directly on spermatogonia in vivo or indirectly via other cell types. To date, we have not further confirmed the various cell types within the testis using techniques such as co-staining with specific markers. Future studies should conduct validation and isolation investigations targeting specific cell types to further elucidate the respective contributions of different cell populations to the protective effect of curcumin against TP-induced testicular injury. While our data demonstrate that both curcumin and Fer-1 independently ameliorate TP-induced injury, the specific mechanism by which curcumin exerts its effects—whether primarily through ferroptosis inhibition or broader antioxidant pathways—remains to be fully elucidated. The combination of curcumin and the ferroptosis inhibitor Fer-1 would provide further insight into this question, which will be a focus of our future investigations. Whether additional ferroptosis-related effector genes are involved requires confirmatory mechanistic studies using gene knockout and overexpression approaches. Whether an autophagy–ferroptosis crosstalk exists in the protective mechanism of curcumin against TP-induced injury to mouse spermatogonia also awaits further investigation. It is worth noting that pre-pubertal boys receiving TP treatment are a particularly vulnerable population, as they are unable to store sperm and their blood–testis barrier is immature, which may lead to increased drug exposure in the testes. Moreover, their developing germ cells are extremely sensitive to injury. Therefore, protective interventions that are safe and effective before puberty are urgently needed. However, our current study used adult mice, and future research in juvenile animal models is necessary to validate the efficacy and safety of curcumin combination therapy during the pre-pubertal stage. Future work should include preclinical toxicology and pharmacokinetic studies to examine the bioavailability of curcumin in vivo, its distribution and metabolism in the testes and spermatogonia, and its impact on spermatogenesis. In addition, the long-term safety of curcumin combined with TP, as well as its potential effects on the reproductive health of offspring, should be explored.

## 5. Conclusions

In summary, curcumin exerted a protective effect against TP-induced injury in mouse spermatogonia. It reduced cell apoptosis, ROS levels, lipid peroxidation, and Fe^2+^ levels, while increasing GSH content. Additionally, in this model system, curcumin regulated ferroptosis-related pathways by modulating the expression of *Nrf2*, *Gclc*, *Map1lc3a*, *Tfrc*, and *Dmt*.

## Data Availability

Data are contained within the article and Supplementary Materials. The relevant data in this study are available from the corresponding author upon reasonable request.

## Supplementary Materials

The following supporting information can be downloaded at: https://www.mdpi.com/article/10.3390/biology15131019/s1, Table S1: Primers used for qRT-PCR; Table S2: Antibodies used for WB and IF experiments; Table S3: Volcano analysis data for Con vs. TP; Table S4: Volcano analysis data for TP vs. Cur-M; Table S5: Clusters of Orthologous Groups (COG) analysis data; Table S6: Subcellular localization analysis data; Table S7: GO annotation enrichment analysis data; Table S8: KEGG pathway analysis data for Con vs. TP; Table S9: KEGG pathway analysis data for TP vs. Cur-M; Table S10: Details of differentially expressed proteins (TP vs. Cur-M) of the ferroptosis pathway; Figure S1: Schematic diagram of mouse epididymal sperm smear; Supplementary File S1: Basis for the determination of TP and curcumin concentrations; Supplementary File S2: Original Western blot images; Supplementary File S3: Individual graphs for each dataset in Figure 3.

## Abbreviations

The following abbreviations are used in this manuscript:

TP	Triptolide
CUR	Curcumin
MDA	Malondialdehyde
GSH	Glutathione
Nrf2	Nuclear factor erythroid 2-related factor 2
Gclc	Glutamate–Cysteine Ligase Catalytic Subunit
Tfrc	Transferrin receptor 1
Map1lc3a	Microtubule-associated protein 1 light chain 3 alpha
Dmt1	Divalent Metal Transporter 1
ROS	Reactive oxygen species
COG	Clusters of Orthologous Groups
