# Supplementary material for: Curcumin Protects Mouse Spermatogonia from Triptolide-Induced Injury Through Modulation of Ferroptosis-Related Pathways
Source: Biology (Basel). 2026 Jun 26;15(13):1019. doi: 10.3390/biology15131019 (PMC13360325; doi:10.3390/biology15131019)
Supplement: Supplementary file 1 [file biology-15-01019-s001.zip › Supplementary File S1. Basis for the determination of TP and curcumin concentrations.pdf]

Supplementary Document S1. Basis for the determination of TP and curcumin concentrations

1. Effects of different doses of triptolide (TP) on spermatogonial injury in mice

Mouse spermatogonial cells (GC-1) were cultured in vitro and passaged, then treated with TP at concentrations of 50 nM, 100 nM, and 200 nM for 24 hours. Cell viability was assessed using trypan blue staining. As shown in Figure 1A, TP treatment led to a marked reduction in cell viability. The CCK-8 assay revealed a significant decrease in cell proliferation (Figure 1B). Annexin V-FITC/PI staining followed by flow cytometry analysis showed that TP treatment substantially increased apoptosis (Figures 1C and 1D). Reactive oxygen species (ROS) levels in GC-1 cells were further measured and found to increase significantly with rising TP concentrations (Figure 2). These results indicate that TP induces oxidative damage in GC-1 cells and ultimately leads to apoptosis.

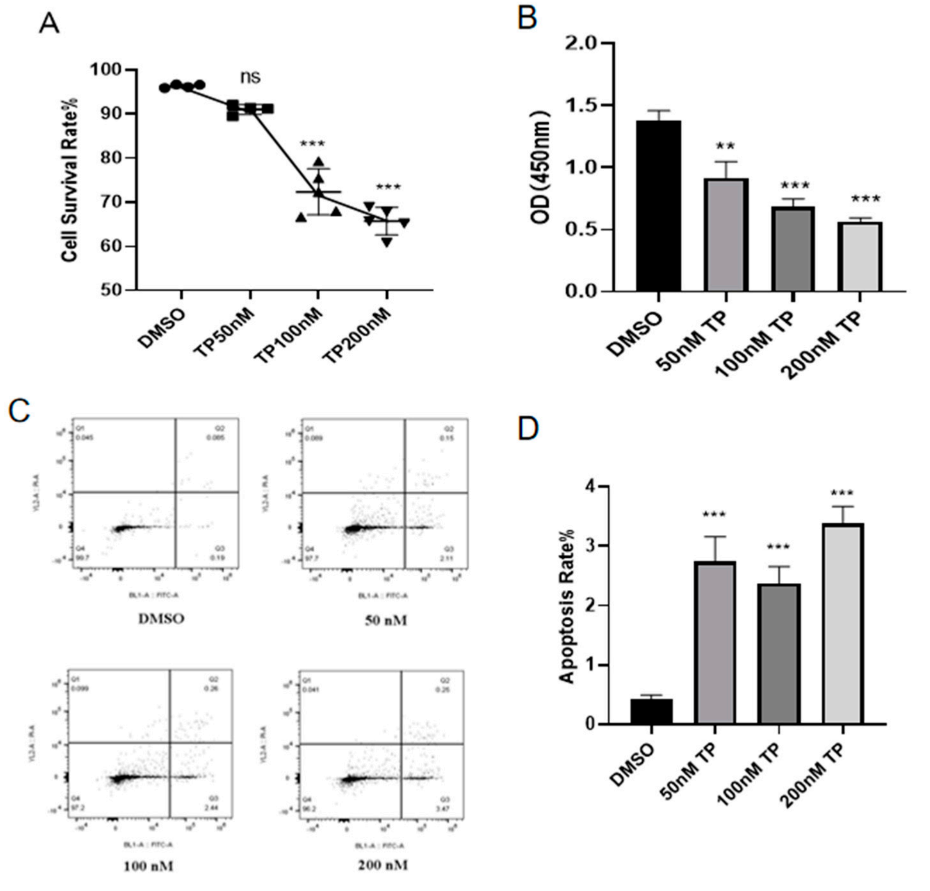

Figure 1. Effect of triptolide (TP) on apoptosis in GC1 cells. (A) Cell viability assessed using trypan blue staining; (B) Cell proliferation determined by CCK-8 assay; (C) Annexin V-FITC/PI staining for apoptosis detection; (D) Quantitative results of apoptosis. *$p < 0.05$, **$p < 0.01$, ***$p < 0.001$.

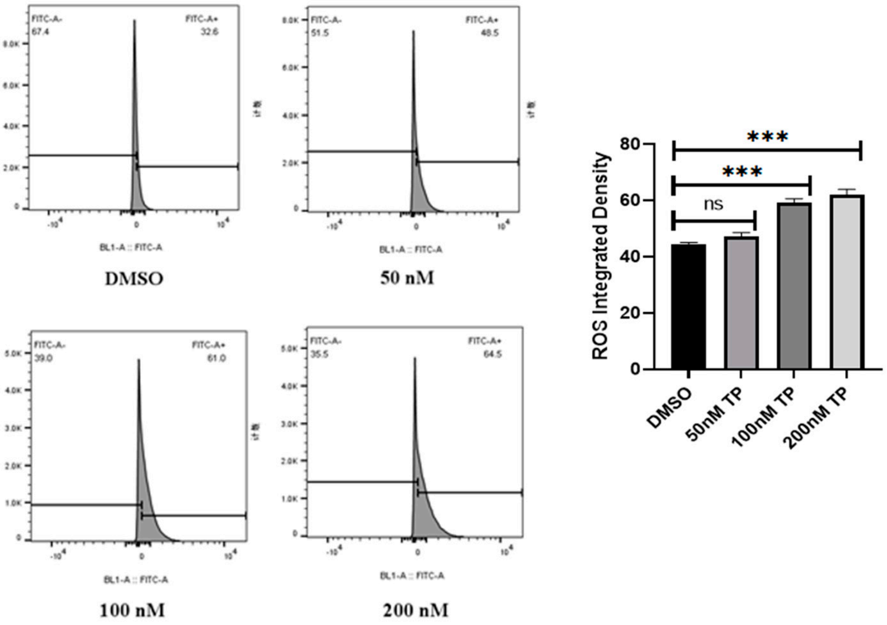

Figure 2. Measurement of reactive oxygen species levels in GC-1 cells. ns, not significant; *p < 0.05, ***p < 0.001.

2. Effects of different doses of curcumin on mouse spermatogonial cells

Mouse spermatogonial cells (GC-1) were cultured in vitro and passaged. A range of curcumin concentrations (2, 4, 6, 8, 10, 12, 14, 16, 18, and 20 μM) was selected based on published literature. After 24 hours of treatment, the CCK-8 assay revealed no significant cytotoxicity at curcumin concentrations below 8 μM. Accordingly, curcumin at 2, 4, and 6 μM was chosen for subsequent experiments.

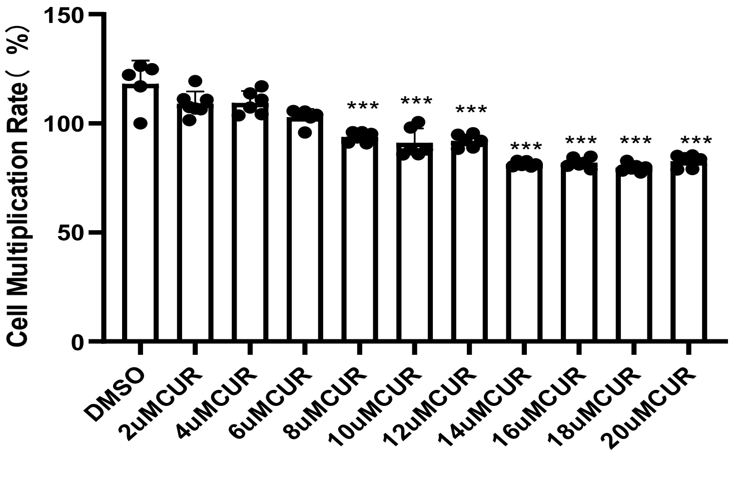

Figure 3. Cell proliferation measured by CCK-8 assay. Data are shown relative to the DMSO group. *p < 0.05, **p < 0.01, ***p < 0.001.

3. Effects of different doses of curcumin on TP‑induced impairment of proliferation in mouse spermatogonial cells

In a preliminary experiment, we investigated the protective effect of varying concentrations of curcumin against triptolide (TP)‑induced damage in the mouse spermatogonial cell line GC1. Cells were co‑treated with TP at 100, 200, or 400 nM and curcumin at 2, 4, or 6 μM (low, medium, and high doses) for 12, 24, or 48 hours. After 24 hours of treatment with 200 nM TP, the cell proliferation rate was significantly lower than that of the control group. Compared with the 200 nM TP group, curcumin‑treated groups showed a marked improvement in proliferation. Co‑treatment with curcumin and either 100 nM or 400 nM TP resulted in no significant improvement (Figure 4).

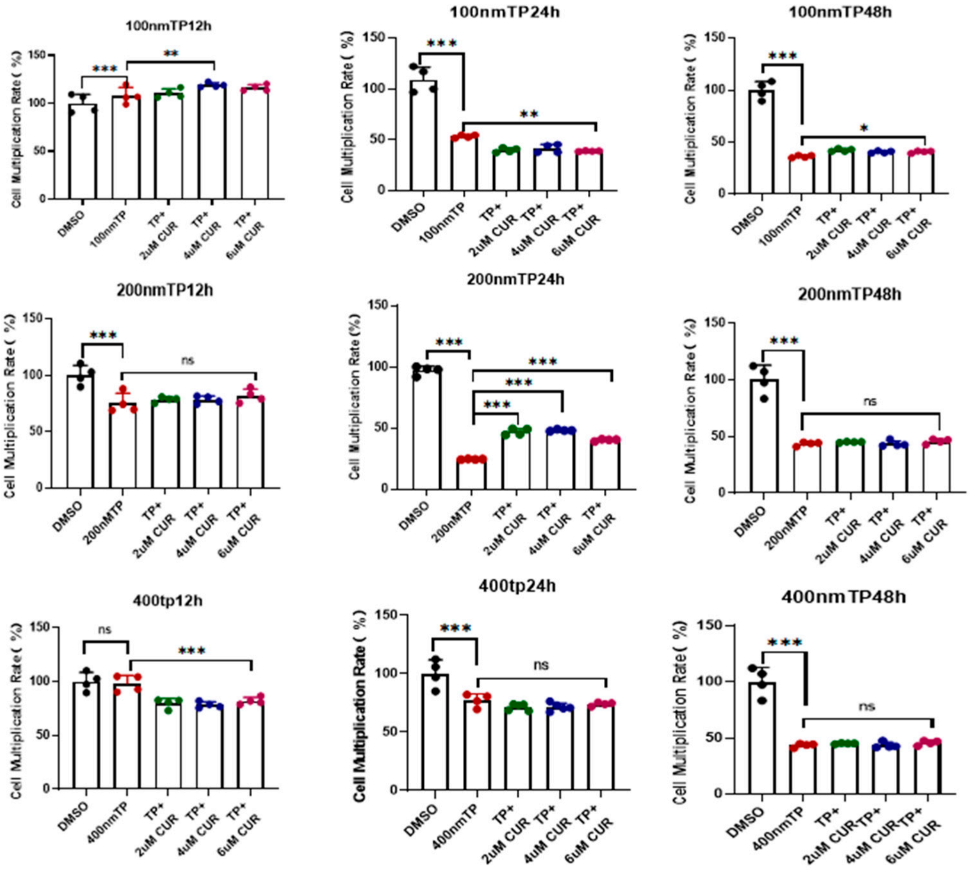

Figure 4. Cell proliferation measured by CCK‑8 assay. ns, not significant; *$p < 0.05$, **$p < 0.01$, ***$p < 0.001$.

4. Literature reference for in vivo concentrations of TP

[1] Wang Y, Xiong B, Chen D, Li J, He Q, Cao Y, et al. Triptolide impairs glycolysis by suppressing GATA4/Sp1/PFKP signaling axis in mouse Sertoli cells. Toxicology and Applied Pharmacology. 2021;426:115606.

[2] Zhao Z, Zhou R, Zhang Y, Jiang H, Zhang H, Liu Q, et al. Triptolide Causes

Spermatogenic Disorders by Inducing Apoptosis in the Mitochondrial Pathway of Mouse Testicular Spermatocytes. Toxics. 2024;12(12):896.

[3] Li J, Chen D, Suo J, Wang Y, He Q, Zhang H, et al. Triptolide induced spermatogenesis dysfunction via ferroptosis activation by promoting K63-linked GPX4 polyubiquitination in spermatocytes. Chemico-Biological Interactions. 2024;400:111130.

[4] Yang X, He L, Zhang Y, Zha X, Wang Y, Liu Q, et al. Triptolide exposure triggers testicular vacuolization injury by disrupting the Sertoli cell junction and cytoskeletal organization via the AKT/mTOR signaling pathway. Ecotoxicology and Environmental Safety. 2024;279:116502.

[5] Qian L, Li Q, Li H, Wang Y, He Q, Wang Y, et al. Triptolide induces Sertoli cell apoptosis in mice via ROS/JNK-dependent activation of the mitochondrial pathway and inhibition of Nrf2-mediated antioxidant response. Acta Pharmacologica Sinica. 2018;39:311－327

5. Literature reference for in vivo concentrations of curcumin

[1] Azizi A, Mohammadi-Sardoo M, Sharififar F, Zeinali M, Pardakhty A, Iranpour M, et al. Comparative evaluation of native and liposomal curcumin against acute reproductive toxicity induced by cadmium chloride in male mice. Andrologia. 2024;2024:6658407. DOI: 10.1155/2024/6658407

[2] Malekshahi Fard N, Khorsandi L, Talebpour Amiri F, et al. Nanocurcumin decreases nucleotide-binding oligomerization domain-like receptor family pyrin domain-containing 3 complex expressions in an experimental testicular torsion model. Int J Fertil Steril. 2024;18(4):411-416. DOI: 10.22074/ijfs.2024.2008608.1511

[3] Wang Z, Chen F, Li Y, Liu C, Wang L, Shao W, et al. Curcumin promotes spermatogenesis in mice with cryptorchidism by regulating testicular protein O-GlcNAcylation. Front Endocrinol. 2025;16:1555721. DOI: 10.3389/fendo.2025.1555721

[4] Khorsandi L, Mirhoseini M, Mohamadpour M, Orazizadeh M, Khaghani S. Effect of curcumin on dexamethasone-induced testicular toxicity in mice. Pharm Biol. 2013;51(2):206-212. DOI: 10.3109/13880209.2012.716854

[5] Shaneh M, Chahardori M, Talebpour Amiri F, Amani N, Shaki F. Mitochondrial protection and anti-inflammatory effect of curcumin in inhibiting reproductive toxicity induced by sodium valproate in male mice. Iran J Basic Med Sci. 2025;28(8):1027-1036. DOI: 10.22038/ijbms.2025.82254.17791
