# Supplementary material for: Curcumin Protects Mouse Spermatogonia from Triptolide-Induced Injury Through Modulation of Ferroptosis-Related Pathways
Source: Biology (Basel). 2026 Jun 26;15(13):1019. doi: 10.3390/biology15131019 (PMC13360325; doi:10.3390/biology15131019)

Supplementary Document S2.Original Western blot images

Migration position variations were noted for certain bands, which could be attributed to factors including gel composition and electrophoresis conditions.

1. Western blot (WB) detection results in GC-1 cells

Nrf2(68kDa)

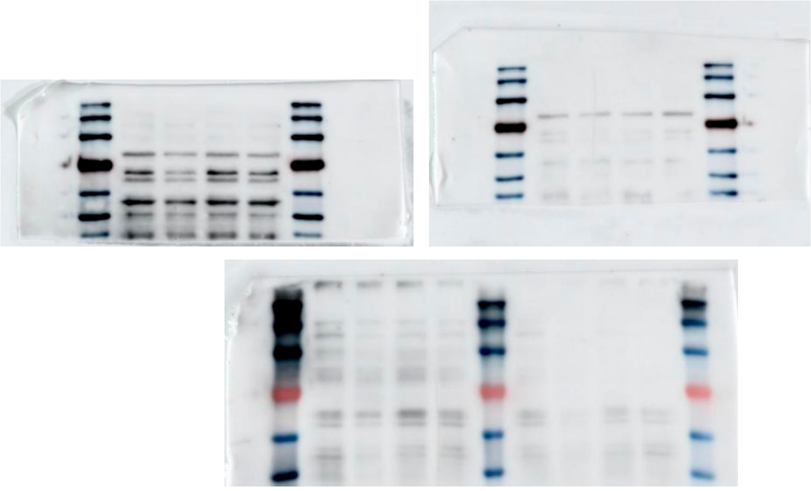

GCLC(73kDa)

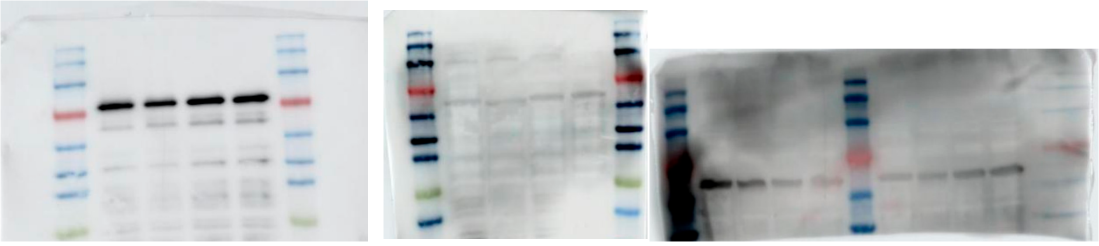

MAP1LC3A(14kDa)

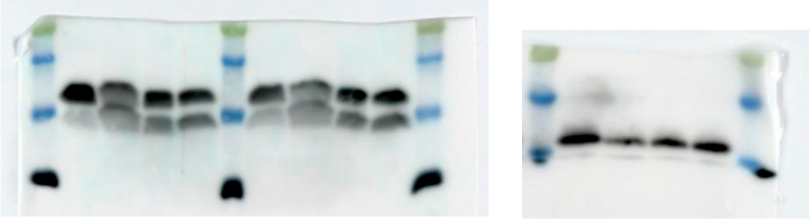

TFRC(74kDa)

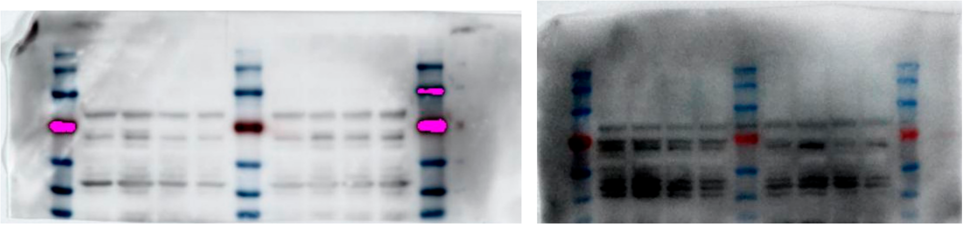

DMt1(62kDa)

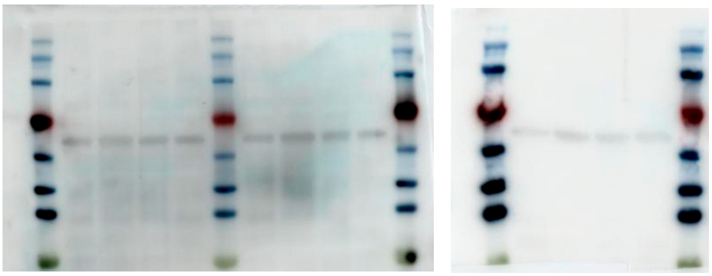

β-actin（42kDa）

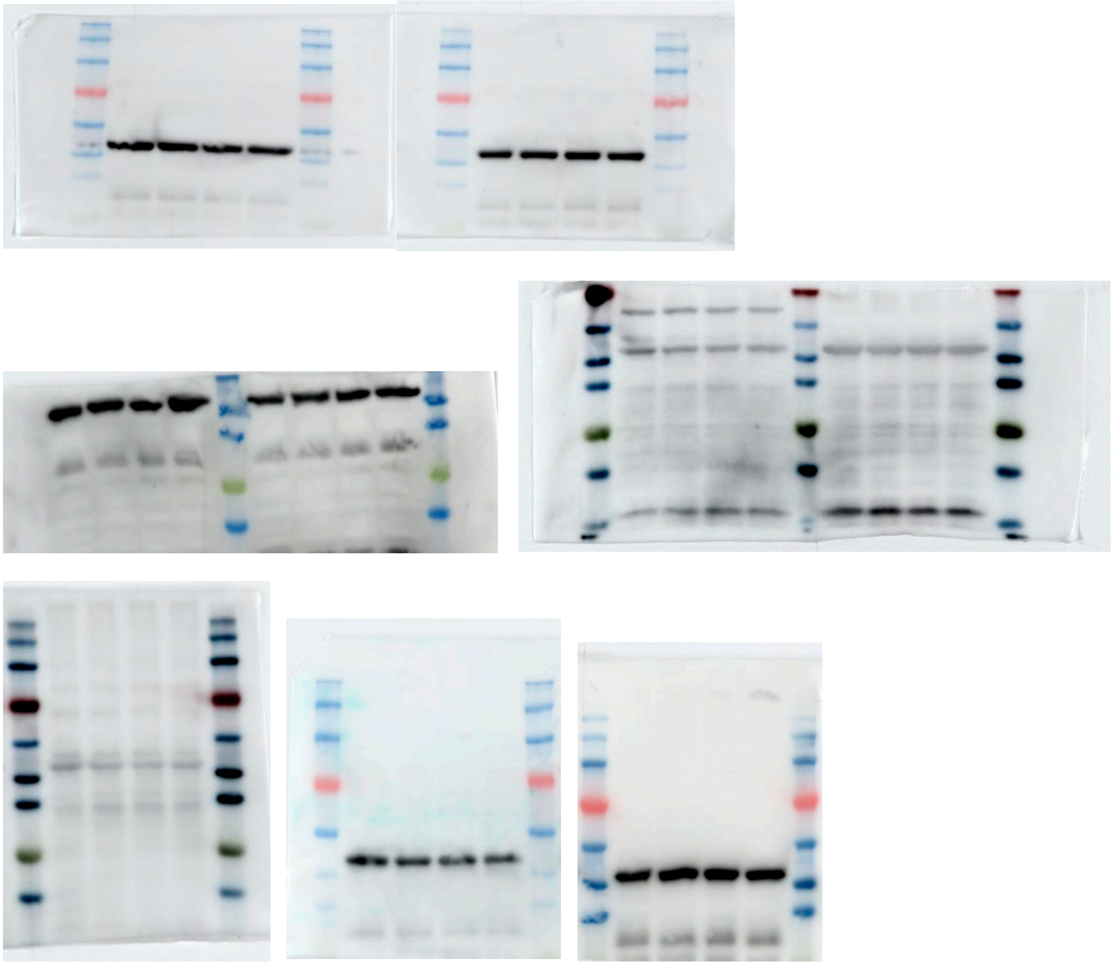

2. Western blot (WB) detection results in mouse testes

Nrf2(68kDa)

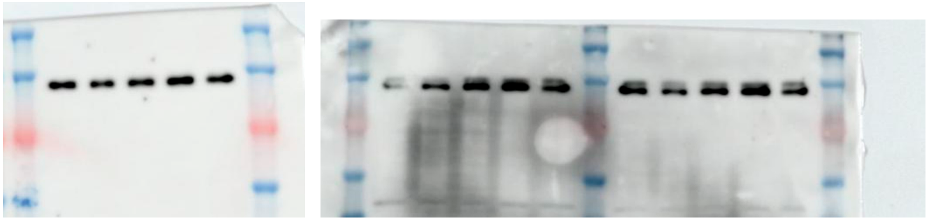

GCLC(73kDa)

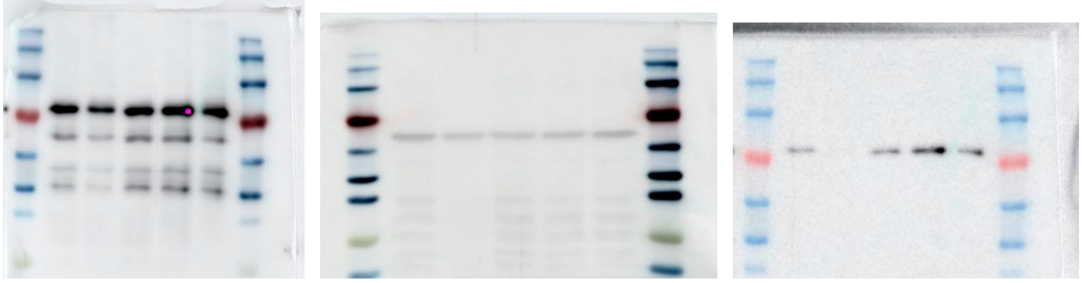

MAP1LC3A(14kDa)

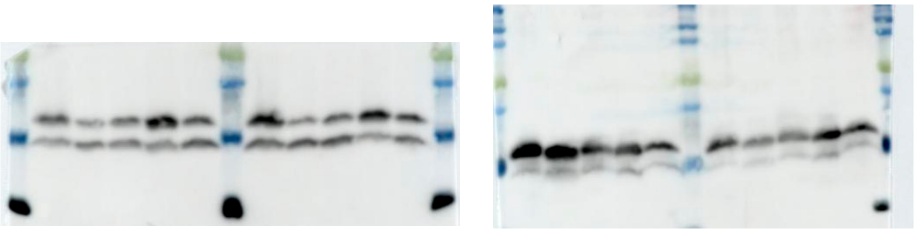

TFRC(74kDa)

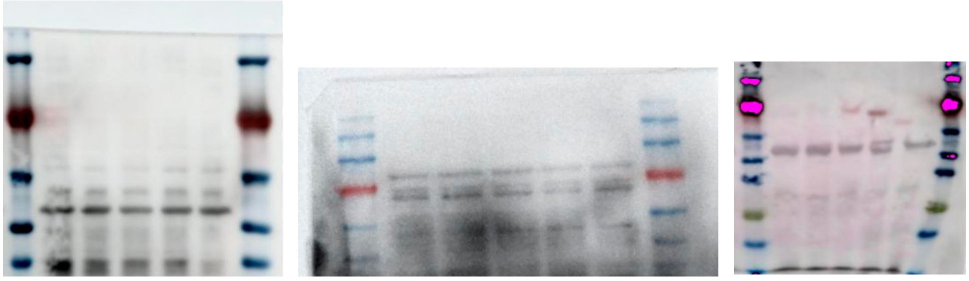

DMt1(62kDa)

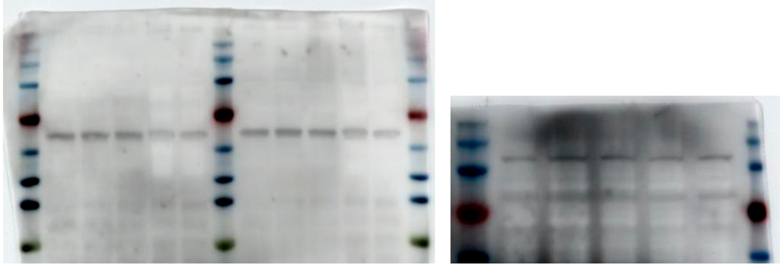

β-actin（42kDa）

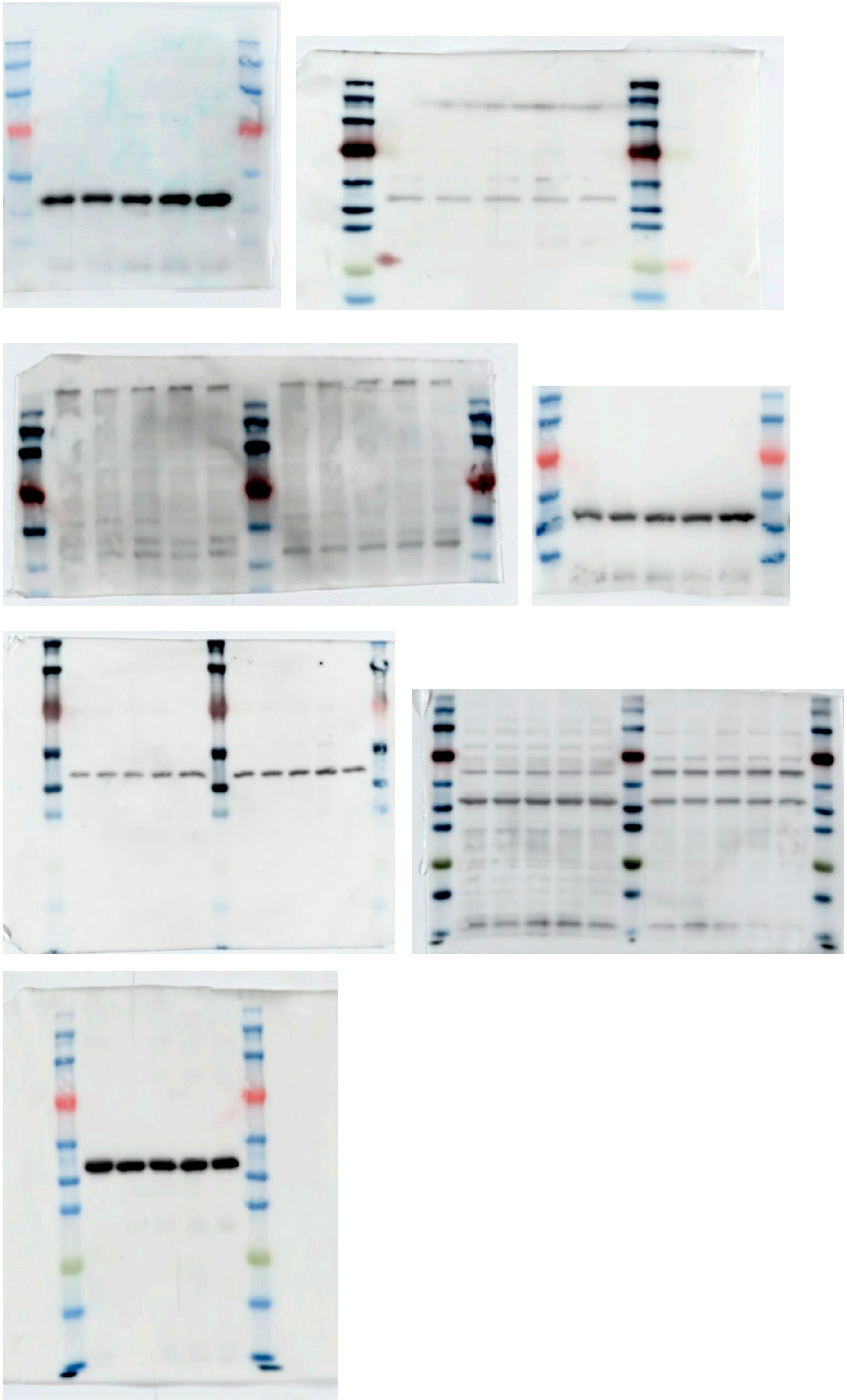

Supplement: Supplementary file 1 [file biology-15-01019-s001.zip › Supplementary File S2. Original Western blot images.pdf]
