# Supplementary material for: Curcumin Protects Mouse Spermatogonia from Triptolide-Induced Injury Through Modulation of Ferroptosis-Related Pathways
Source: Biology (Basel). 2026 Jun 26;15(13):1019. doi: 10.3390/biology15131019 (PMC13360325; doi:10.3390/biology15131019)
Supplement: Supplementary file 1 [file biology-15-01019-s001.zip › Supplementary File S3. Individual graphs for each dataset in Figure 3.pdf]

Supplementary Document S3.Individual graphs for each dataset in Figure 3.

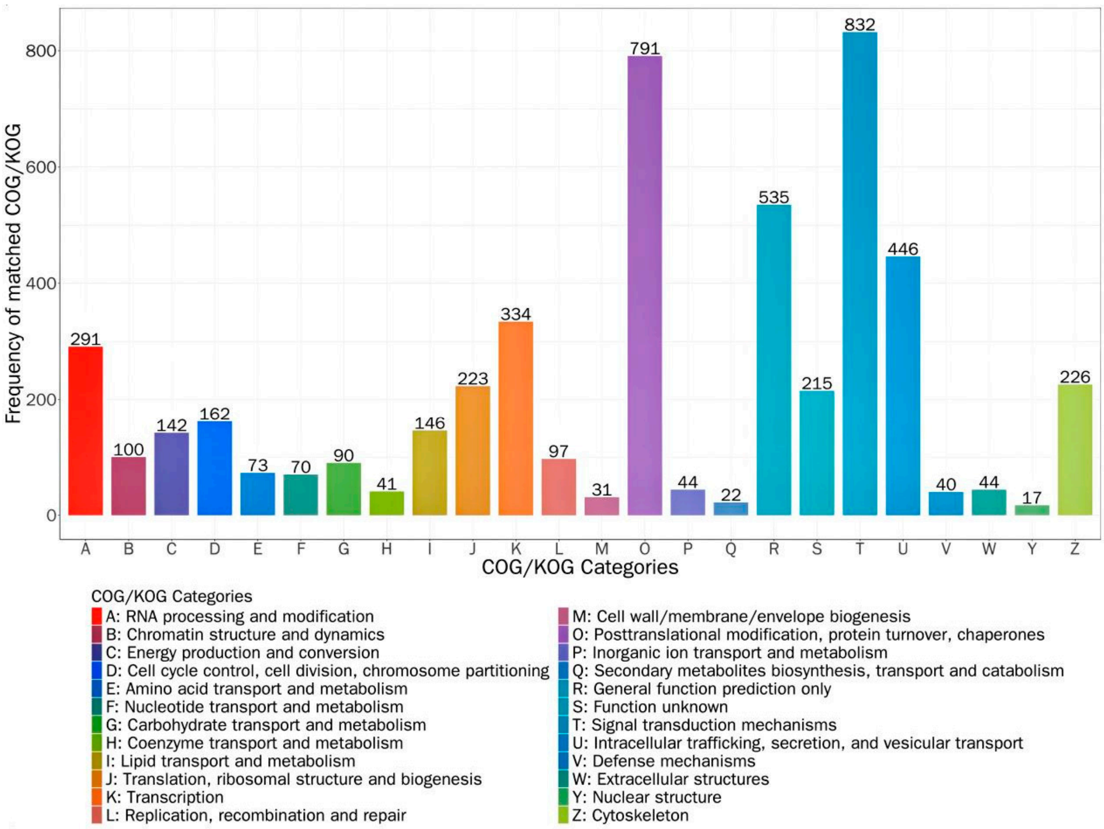

(A) Histogram of COG analysis for differentially expressed proteins: The horizontal axis represents the functional categories of COG classification, and the vertical axis indicates the frequency of COG assignments.

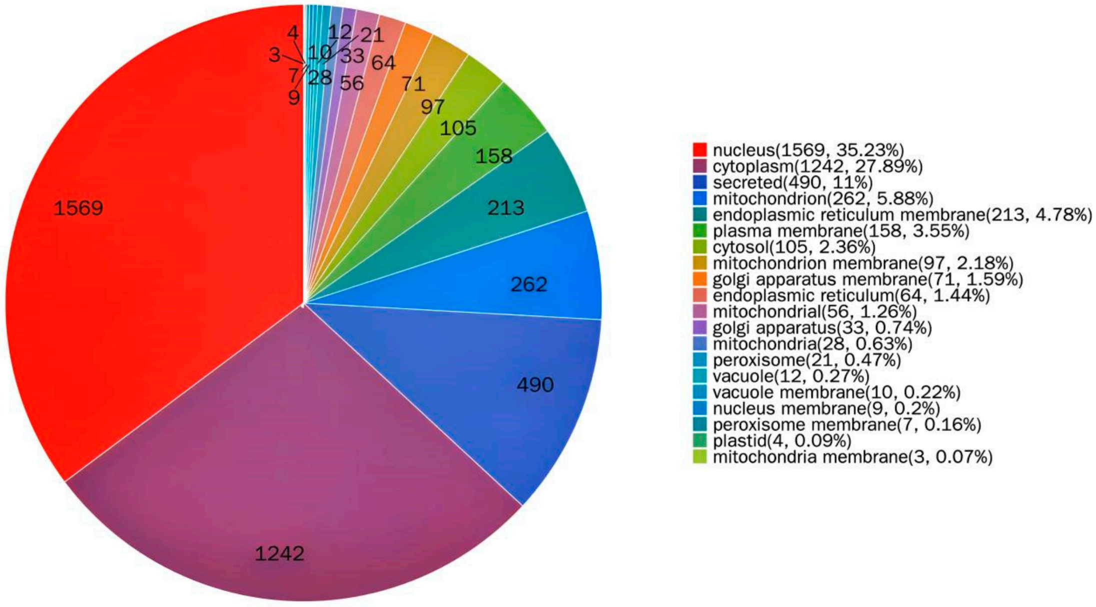

(B) Pie chart illustrating the subcellular localization analysis of differentially expressed proteins.

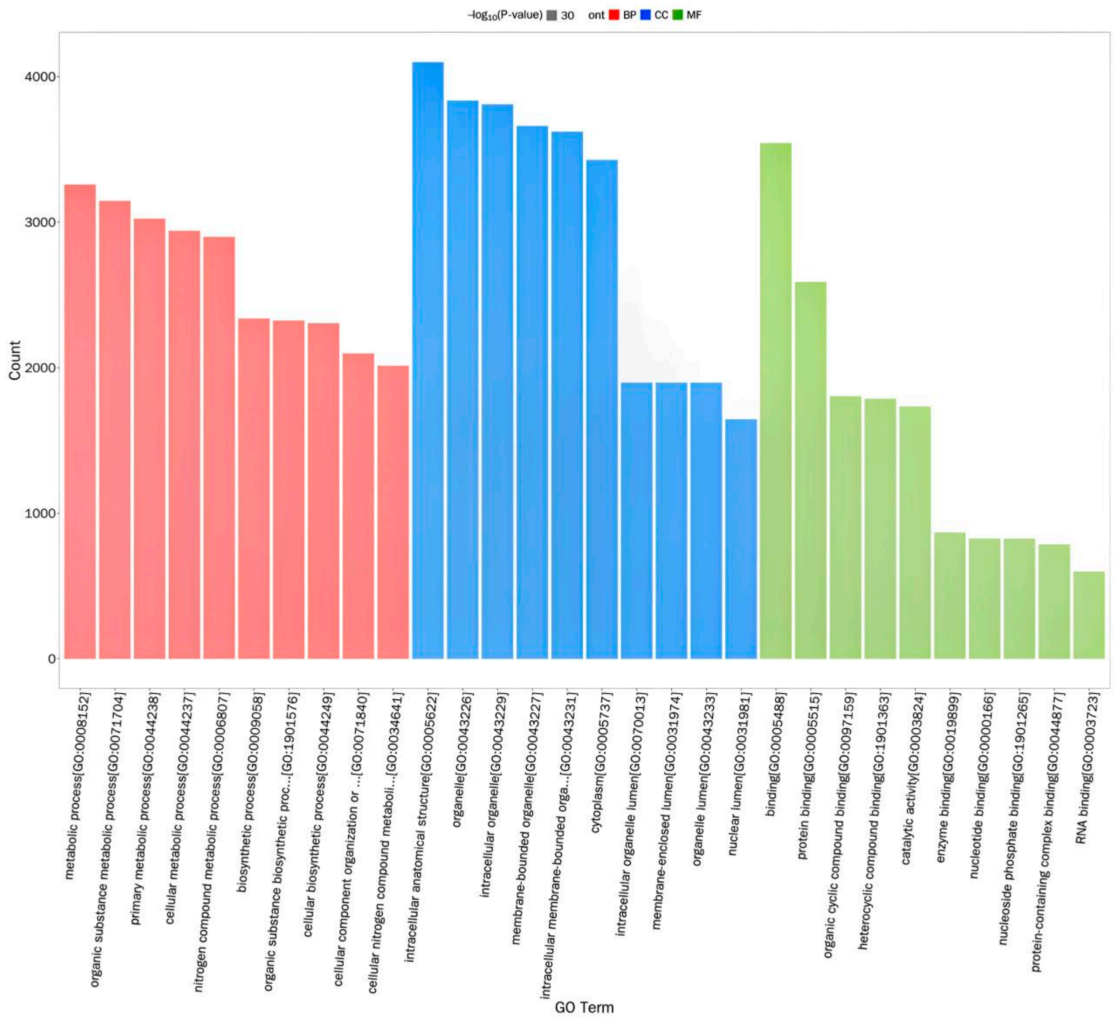

(C) Bar chart of GO enrichment analysis for the comparison Con vs Tp vs Cur-M.

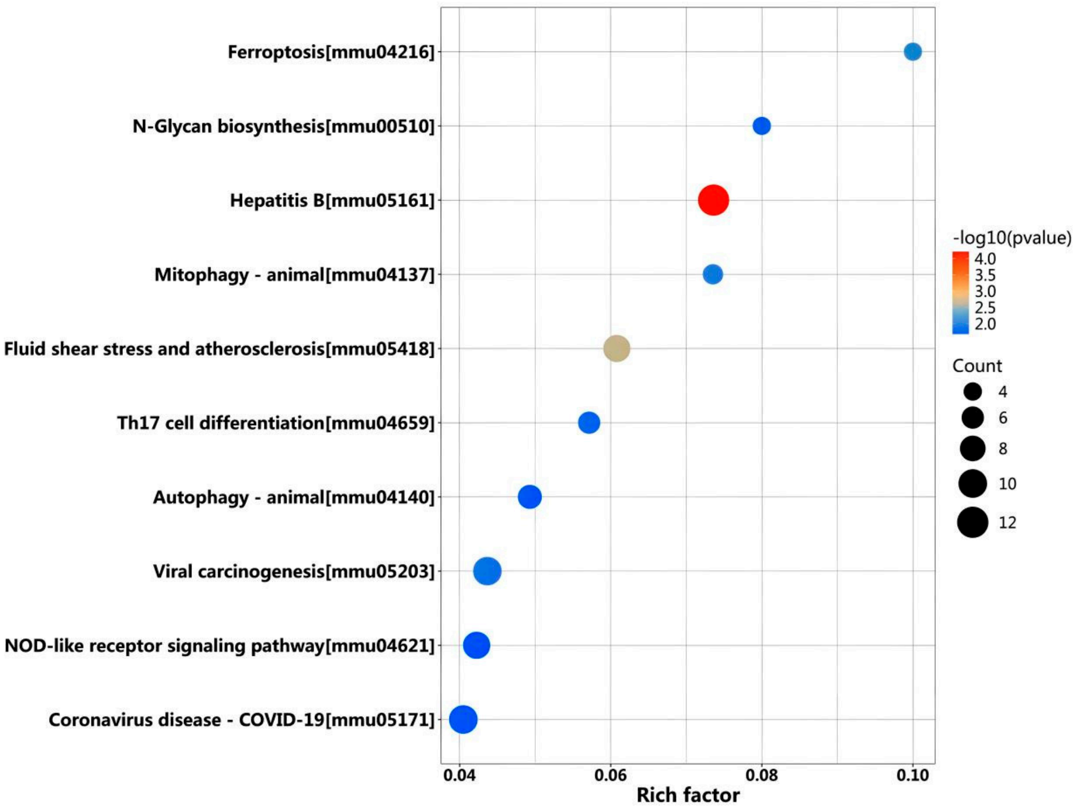

(D) Bubble plot of KEGG pathway enrichment analysis for the comparison Con vs Tp.

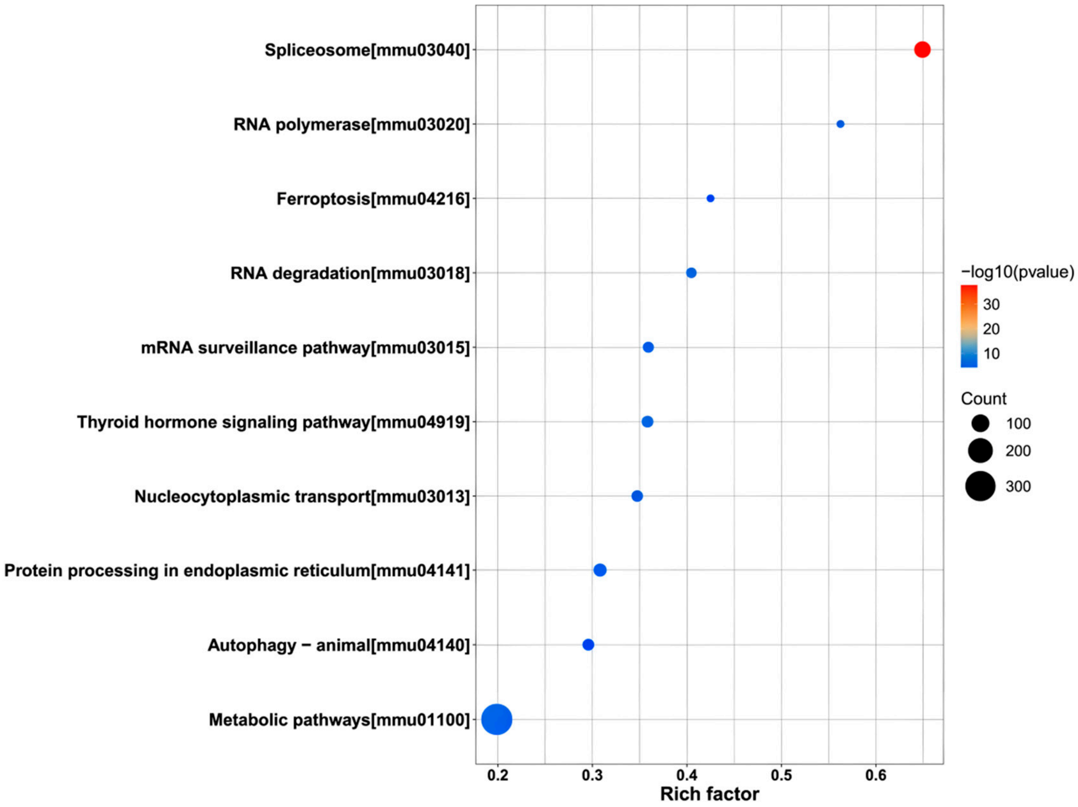

(E) Bubble plot of KEGG pathway enrichment analysis for the comparison Tp vs Cur-M.
