# Supplementary figures and images for: Curcumin Protects Mouse Spermatogonia from Triptolide-Induced Injury Through Modulation of Ferroptosis-Related Pathways
Source: Biology (Basel). 2026 Jun 26;15(13):1019. doi: 10.3390/biology15131019 (PMC13360325; doi:10.3390/biology15131019)

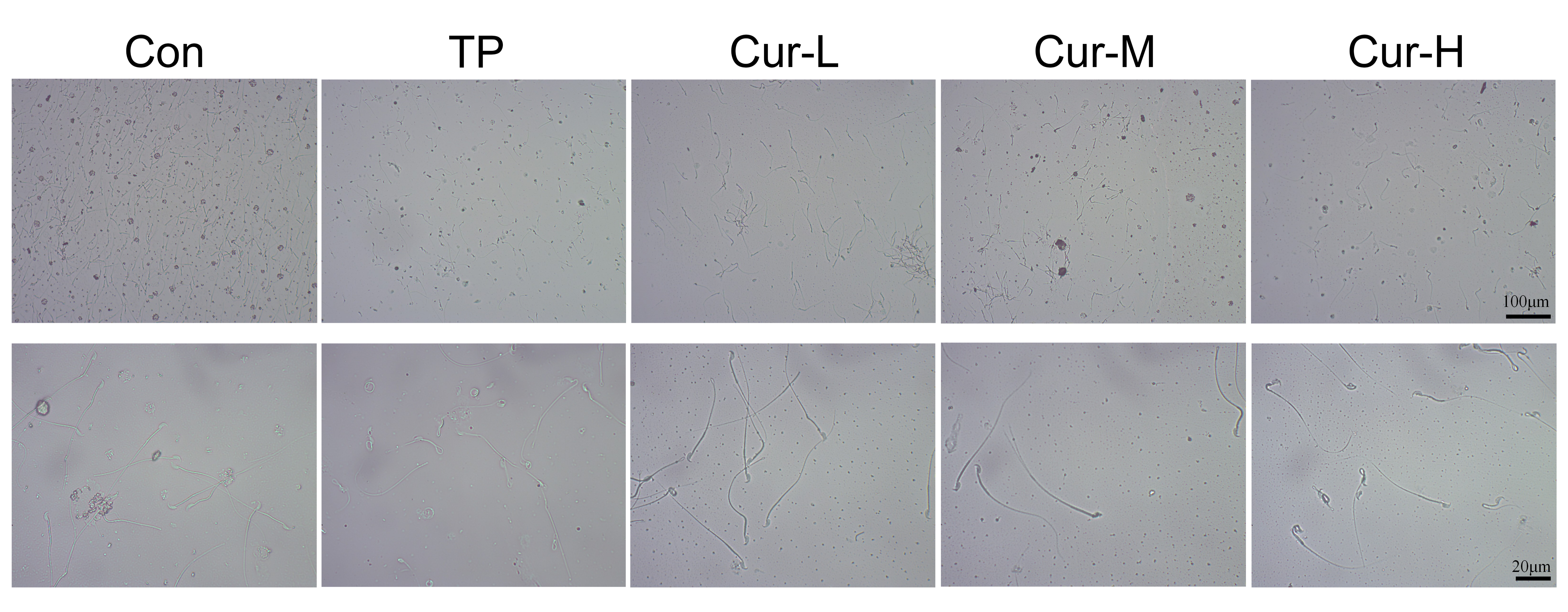

Supplement: Supplementary file 1 [file biology-15-01019-s001.zip › Figure S1. Schematic diagram of mouse epididymal sperm smear.png]
